# Hypoxia-induced NLRP3 inflammasome activation via the HIF-1α/NF-κB signaling pathway in human dental pulp fibroblasts

**DOI:** 10.1186/s12903-024-04936-w

**Published:** 2024-09-29

**Authors:** Diya Wang, Minghao Wang, Shukai Sun, Chongyang Zhang, Ya Song, Jianing Li, Bing Song, Haipeng Lv, Shengchao Wang, Wenkai Jiang

**Affiliations:** 1 State Key Laboratory of Oral & Maxillofacial Reconstruction and Regeneration & National Clinical Research Center for Oral Diseases & Shaanxi Key Laboratory of Stomatology, Department of Operative Dentistry & Endodontics, School of Stomatology, Fourth Military Medical University, No.145 Western Changle Road, Xi’an, Shaanxi 710032 China; 2 Department of Occupational and Environmental Health and the Ministry of Education Key Lab of Hazard Assessment and Control in Special Operational Environment, School of Public Health, Fourth Military Medical University, Xi’an, Shaanxi China; 3 State Key Laboratory of Oral & Maxillofacial Reconstruction and Regeneration & National Clinical Research Center for Oral Diseases & Shaanxi Clinical Research Center for Oral Diseases, Department of Pediatric Dentistry, School of Stomatology, Fourth Military Medical University, Xi’an, Shaanxi China; 4 School of Dentistry, College of Biomedical and Life Sciences, Cardiff University, Cardiff, CF14 4XY UK; 5 Shenzhen Institutes of Advanced Technology, Chinese Academy of Sciences, Shenzhen, 518055 China; 6 Department of Stomatology, Xi’an Daxing Hospital, Xi’an, Shaanxi China

**Keywords:** NLRP3, HIF-1α, Human dental pulp fibroblasts, Pulpitis, Sterile inflammation

## Abstract

**Background:**

Previous studies have reported the link between hypoxic conditions and NLRP3 inflammasome-mediated pulpal inflammation in the progression of pulpitis. However, the underlying mechanism has not been fully elucidated. This study aimed to investigate the role of HIF-1α in the regulation of NLRP3 inflammasome pathway via NF-κB signaling under hypoxic conditions with or without LPS in human dental pulp fibroblasts (HDPFs) during the progression of pulpitis.

**Methods:**

HIF-1α plasmids or siRNAs were used to upregulate or downregulate HIF-1α in HDPFs, respectively. The effect of hypoxia with or without LPS on the NF-κB signaling and NLRP3 inflammasome pathway was analyzed by immunofluorescence staining, qRT-PCR, western blotting and ELISA.

**Results:**

The hypoxic conditions alone induced ASC oligomerization and NLRP3/CASP1 inflammasome pathway activation via NF-κB signaling in a time-dependent manner in HDPFs. The upregulation of HIF-1α further promoted hypoxia-induced ASC oligomerization and NLRP3/CASP1 inflammasome pathway activation via NF-κB signaling compared to the hypoxia-induced group. In comparison, downregulation of HIF-1α inhibited ASC oligomerization and NLRP3/CASP1 inflammasome pathway activation via NF-κB signaling compared to the hypoxia-induced group. Additionally, LPS plus hypoxia further promoted HIF-1α expression and NLRP3/ASC/CASP1 inflammasome pathway activation via NF-κB signaling compared to the hypoxia-induced group.

**Conclusions:**

HIF-1α served as a positive regulator of NLRP3/ASC/CASP1 inflammasome pathway activation via NF-κB signaling in HDPFs in the sterile pulpal inflammation and caries-related pulpitis microenvironment. The finding of a novel functional HIF-1α-NF-κB-NLRP3 axis provides insight into the link between the hypoxic microenvironment and pulpal inflammation, thus supporting a promising therapeutic strategy for the control of pulpal inflammation.

**Supplementary Information:**

The online version contains supplementary material available at 10.1186/s12903-024-04936-w.

## Introduction

Pulpitis caused by caries-related microorganisms is one of the most prevalent infectious diseases in the world [1]. Intradentinal penetration of cariogenic microorganisms and the diffusion of microbial byproducts through dentin tubules into the pulpal tissue are responsible for pulp inflammation initiation and progression [2]. Additionally, sterile inflammation can also occur in pulpal tissue because of dental trauma or orthodontic force application [3]. Due to the encasement of pulp tissue within rigid dentine walls, dental pulp is trapped in low oxygen conditions and low glucose conditions when circulatory impairment is induced by the initiation of inflammation [4]. Notably, several studies have highlighted a strong link between the hypoxic microenvironment and inflammation [5–7]. However, whether the hypoxic microenvironment promotes the progression of pulpitis and its underlying molecular mechanism has yet to be determined. The investigation of mechanisms of dental pulp response to hypoxic microenvironment may provide a promising treatment strategy for controlling the progression of pulpitis.

As a subunit of the heterodimeric transcription factor hypoxia-inducible factor 1 (HIF1), HIF-1α is a key transcription factor in hypoxia that contributes to the regulation of energy metabolism, the maintenance of hematopoietic stem cells, angiogenesis, proliferation, apoptosis, invasion, and metastasis of cancer cells [8–11]. In the hypoxic microenvironment, prolyl hydroxylase activity is inhibited. This inhibition leads to the translocation and accumulation of stable HIF-1α subunits in the nucleus, where they bind with HIF-1β and participate in multiple signal transduction pathways, which are associated with many pathological conditions, particularly inflammatory diseases [12–14]. Hypoxic conditions accelerate the release of proinflammatory cytokines, which may be important in exacerbating the inflammatory response [15, 16]. However, the role of HIF-1α in the regulation of proinflammatory cytokine release, which leads to the pathophysiological process of pulpal inflammation, is still unclear.

The NLR family, pyrin domain containing 3 (NLRP3) is composed of NLRP3, ASC, and caspase-1 that can initiate inflammatory reactions in response to pathogenic infections and tissue injury [17]. Human dental pulp fibroblasts (HDPFs), the main cells of dental pulp, play a crucial role in controlling pulp vascularization, inflammation/ immune response, and regeneration [18]. Previous studies have reported that activation of the NLRP3/CASP1 inflammasome pathway in HDPFs promotes the progression of pulpitis [19, 20]. We found the upregulation of HIF-1α accompanied by the activation of the NLRP3 inflammasome during the development from reversible to irreversible pulpitis, which indicated the link between hypoxic conditions and pulpal inflammation [21, 22]. However, during pulpal sterile inflammation, whether hypoxia induction can directly activate the NLRP3 inflammasome and the role of HIF-1α in regulating NLRP3/CASP1 inflammasome pathway activation in HDPFs remain unclear. Additionally, during caries-related pulpitis, bacterial infection is naturally accompanied by a hypoxic microenvironment, and both factors are highly connected and important [23]. Therefore, this study aimed to investigate the effect of hypoxia with or without LPS on the NLRP3 inflammasome, as well as the role of HIF-1α in the regulation of the NLRP3 inflammasome pathway, which may be helpful in screening potential therapeutic targets for the control of the development of sterile pulpitis caused by trauma as well as caries-related pulpitis.

## Materials & methods

### Cell cultures

The pulp tissues were obtained and HDPFs were isolated as previously described after patient consent and ethical approval by the ethics committee of the School of Stomatology, Fourth Military Medical University (permission number IRB-REV-2017–007) [19]. HDPFs at the third or fourth passages were used for this study. For the normoxic condition, HDPFs were cultured at 37℃, a gas mixture of 21% O_2_, 5% CO_2_ and 74% N_2_. For the hypoxic condition, HDPFs were cultured at 37℃, a gas mixture of 1% O_2_, 5% CO_2_ and 94% N_2_ [24]. ATP and LPS (from Escherichia coli serotype O111:B4) were purchased from Sigma-Aldrich (USA) [19]. For the ATP plus LPS induction experiments, HDPFs were incubated with ATP (5 mM) for 2 h, and then exposed to LPS (10 μg/ml) for 6 h as previously reported [21].

### Reverse transcriptase PCR (RT-PCR) and qRT-PCR

Total RNA was extracted from the HDPFs using an RNeasy Mini Kit (QIAGEN, Crawley, UK) according to manufacturer’s instructions. The RT-PCR was performed with RT-PCR kit (Promega, Southampton, UK). The qRT-PCR was performed with the SYBR Green PCR master mix reagent (Takara, Otsu, Japan). The protocol of RT-PCR and qRT-PCR referred to the previous published study [22]. The human gene-specific primers for cDNA were shown in Table 1.

**Table 1:** Primer sequences

Genes	Forward and reverse primers	Accession number
GAPDH	5'-GCACCGTCAAGGCTGAGAAC-3' 5'-TGGTGAAGACGCCAGTGGA-3'	NM_002046.3
CD146	5'-CATCGTGGCTGTGATTGTG-3'	NM_006500.3
	5'-TTCTGGGAGCTTATCTGACTTA-3'	
CD29	5'-AATGTAACCAACCGTAGC-3'	NM_002211.4
	5'-CAGGTCCATAAGGTAGTAGAG-3'	
CD90	5'-TAGTGGACCAGAGCCTTCG-3'	NM_006288.3
	5'-TTCGGGAGCGGTATGTG-3'	
CD105	5'-CACTAGCCAGGTCTCGAAGGG-3' 5'-CTGTTTACACTGAGGACCAGAAGC-3'	NM_001114753.2
CD34	5'-GTCTTCCACTCGGTGCGTCTC-3' 5'-GTTCCCTGGGTAGGTAACTCT-3'	NM_001025109.1
CD45	5'-ATTGCGATTTCCGTGTAA-3' 5'-CAAGCAGGGCTATTGATG-3'	NM_001267798.2
HIF-1α	5'- GAAACTTCTGGATGCTGGTG-3' 5'- CAAACTGAGTTAATCCCATG -3'	NM_001243084.1
NLRP3	5'- ATTCGGAGATTGTGGTTGGG-3' 5'- GAGGGCGTTGTCACTCAGGT-3'	XM_017000183.1
IL-1β	5'- GAATCTCCGACCACCACTAC-3' 5'- CACATAAGCCTCGTTATCCC-3'	NM_000576.3
IL-18	5'- AGATAGCCAGCCTAGAGGTA-3' 5'- TTATCAGGAGGATTCATTTC-3'	NM_001243211.2

### Immunocytochemistry

The protocol of immunocytochemistry referred to the previous published study [21]. Primary antibodies were anti-vimentin (1:100, CST, London, UK), anti-keratin (1:100, CST), anti-HIF-1α (1:100, CST), and anti-NLRP3 (1:100, CST). The secondary antibodies were anti-mouse IgG Alexa Fluor-488 or anti-rabbit IgG Alexa Fluor-594 (1:1000, CST).

### DNA construction and cell transfection

For HIF-1α overexpression, the full-length human HIF-1α coding region was amplified by PCR and cloned into pcDNA3.1 (Invitrogen, Carlsbad, USA). SiRNAs targeting HIF-1α were obtained from Ribo Biotechnology Company (Guangzhou, China) and transfected into cells using Lipofectamine 2000 reagent (Invitrogen). Empty vectors of Up NC and Down NC were used as HIF-1α overexpression and downregulation controls, respectively.

### Western blot and ASC oligomerization

The protocol of western blot analysis referred to the previous published study [21]. The ASC oligomerization was performed as described previously [25]. The primary antibodies were HIF-1α (1:1000; CST), NLRP3 (1:500; CST), caspase-1 (1:400; CST), ASC (1:1000; CST), NF-κB p65 (1:1000; CST), NF-κB p-p65 (1:500; CST), IκB-α (1:500; CST), p-IκB-α (1:500; CST) and β-actin (1:400; CST). The secondary antibodies were anti-mouse IgG antibody or anti-rabbit IgG diluted 1: 2000 (CST).

### Enzyme-linked Immunosorbent assay (ELISA)

The cell culture supernatant was collected after centrifugation. The amount of IL-1β and IL-18 protein in the culture medium was quantified using ELISA kits (R&D, Minneapolis, MN, US) according to the manufacturer’s instruction.

### Statistical analysis

Minimal 3 independent experiments are presented as the mean ± SE (standard error). The significance of the differences was determined by one-way analysis of variance (ANOVA) with Tukey's Multiple Comparison Test; *P* < 0.05 indicated statistical significance.

## Results

### Isolation and characterization of HDPFs

**Fig. 1:**
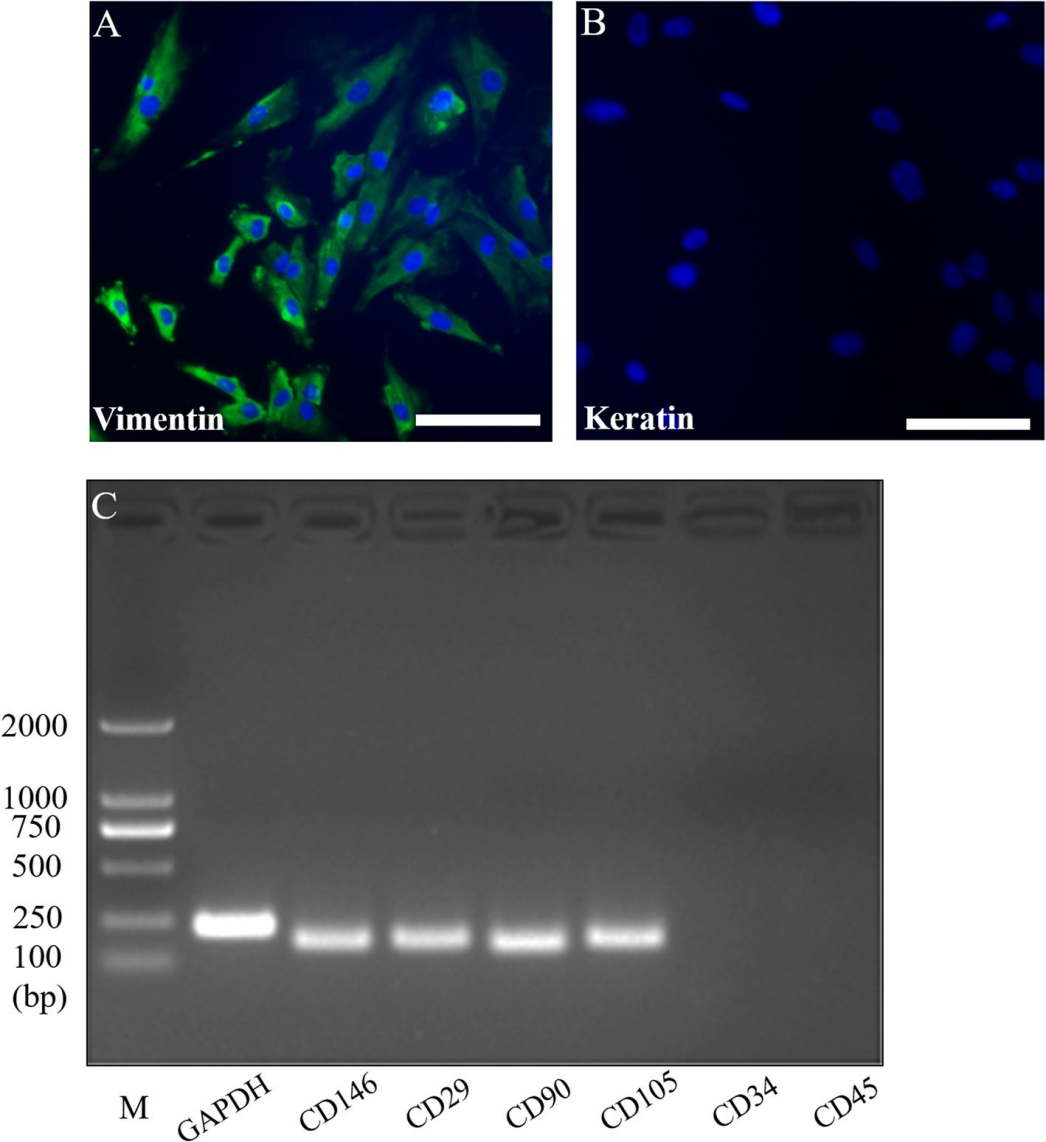
Isolation and characterization of HDPFs. Characterization of HDPFs by immunofluorescence detection and RT-PCR; positive for vimentin **A**; negative for keratin **B**; positive for CD146, CD29, CD90, CD105; negative for CD34 and CD45 **C**. *Bar* 50 μm

HDPFs were successfully isolated from the pulp tissue of the extracted third molars as described previously [22]. HDPFs at the third passages were characterized by immunocytochemical staining and RT-PCR. The results of immunocytochemical staining demonstrated that HDPFs were positive for vimentin and were negative for keratin (Fig. 1A, B). Additionally, the results of RT-PCR revealed that HDPFs positively expressed CD146, CD29, CD90 and CD105, which are mesenchymal cell markers, and negatively expressed CD34 and CD45, which are endothelial cells markers (Fig. 1C).

### Hypoxia activates the HIF-1α, NLRP3 and NF-κB signaling in HDPFs

**Fig. 2:**
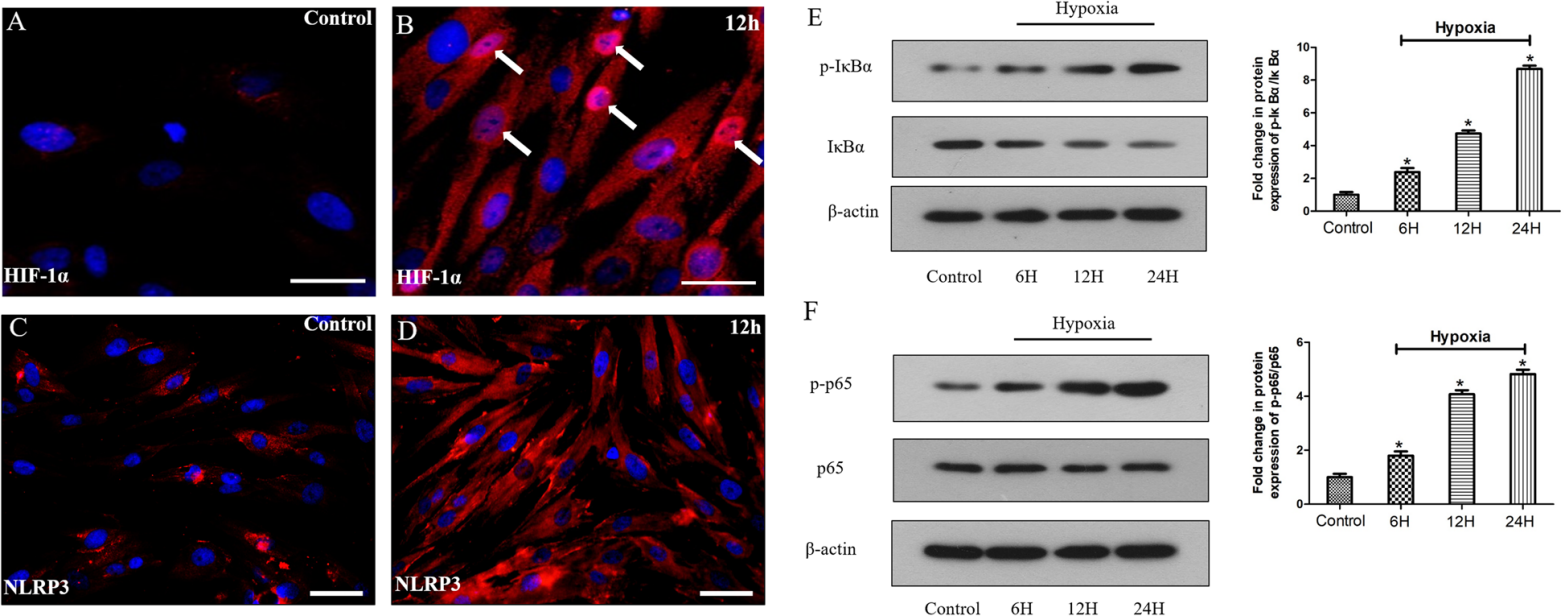
Hypoxia induces the HIF-1α, NLRP3 and NF-κB signaling activation in HDPFs. Immunofluorescence detection of HIF-1α and NLRP3 in HDPFs induced by hypoxia **A**-**D**. The protein levels of IκBα, p-IκBα, p65, p-p65 and β-actin was analyzed by western blotting, and the relative band intensities were determined by densitometry **E**, **F**. Statistical analysis was performed using one-way ANOVA with Tukey's Multiple Comparison Test. *P < 0.05 compared with the control group. Full-length blots/gels are presented in Supplementary Fig. 1–2

The protein expression of HIF-1α and NLRP3 was analyzed by immunocytochemistry in HDPFs induced by hypoxia for 12 h. None of the cells that were cultured under normoxic conditions stained positive for HIF-1α (Fig. 2A). The cells stained positive for HIF-1α in the cytoplasm and nucleus that were induced by hypoxia for 12 h (Fig. 2B). Only a few cells were found to express NLRP3 in the control group (Fig. 2C). Most cells stained positive for NLRP3 after 12 h of hypoxia (Fig. 2D). A time course to detect NF-κB signaling p65 and IκBα protein expression in HDPFs that were induced by hypoxia was examined by western blotting. The phosphorylation of IκBα (p-IκBα) and NF-κB p65 (p-p65) was upregulated and IκBα was downregulated by hypoxia in a time-dependent manner, whilst the protein level of p65 did not change (Fig. 2E, F).

### Hypoxia induces ASC oligomerization and subsequent activation of the NLRP3/CASP1 inflammasome pathway in HDPFs

**Fig. 3:**
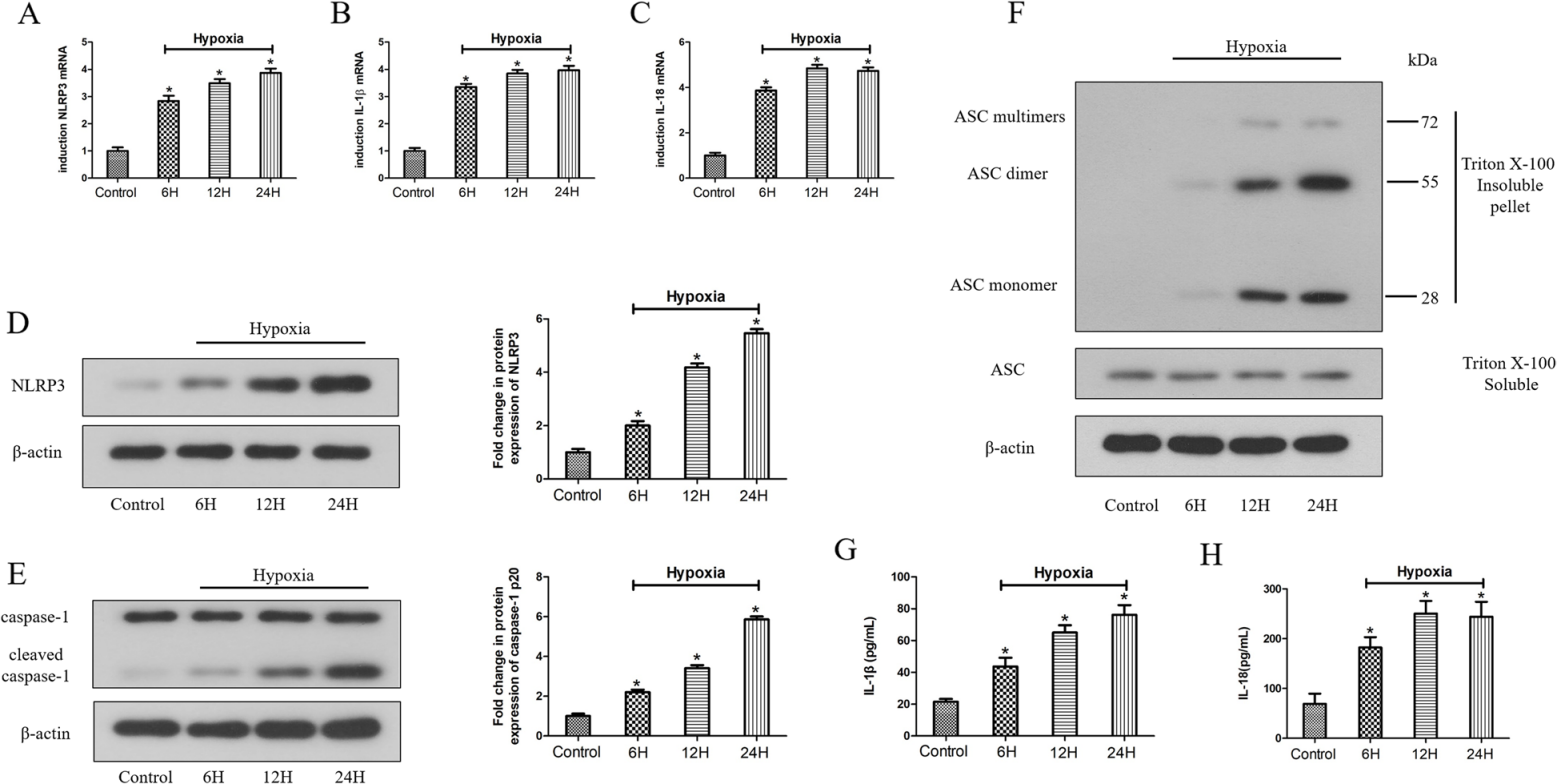
Hypoxia induces ASC oligomerization and subsequent the NLRP3 inflammasome pathway activation in HDPFs. HDPFs induced by hypoxia for a times course. The mRNA and protein expression of NLRP3, caspase-1, ASC, IL-1β and IL-18 were analyzed by qRT-PCR **A-C**, western blotting **D-F** and ELISA **G**, **H**, respectively. Statistical analysis was performed using one-way ANOVA with Tukey's Multiple Comparison Test. *P < 0.05 compared with the control group. Full-length blots/gels are presented in Supplementary Fig. 3–5

The expression of NLRP3, caspase-1, IL-1β and IL-18 was analyzed by the detection of the relevant mRNA and protein in HDPFs induced by hypoxia in a time course using qRT‒PCR, western blotting and ELISA, respectively. Hypoxia induction upregulated NLRP3, IL-1β and IL-18 mRNA expression (Fig. 3A-C), NLRP3 and caspase-1 p20 protein expression (Fig. 3D, E), and exhibited increased secretion of IL-1β and IL-18 with time (Fig. 3G, H). ASC oligomerization plays a key role in the NLRP3 inflammasome activation. ASCs form a dimer or oligomer in inflammasome-activated conditions [17]. Western blot analysis revealed that ASC oligomerization was induced by hypoxia, suggesting that hypoxia can promote ASC oligomerization and subsequent NLRP3/CASP1 inflammasome pathway activation in HDPFs (Fig. 3F).

### Effects of HIF-1α on hypoxia-induced ASC oligomerization and subsequent NLRP3/CASP1 inflammasome pathway activation via the NF-κB signaling in HDPFs

**Fig. 4:**
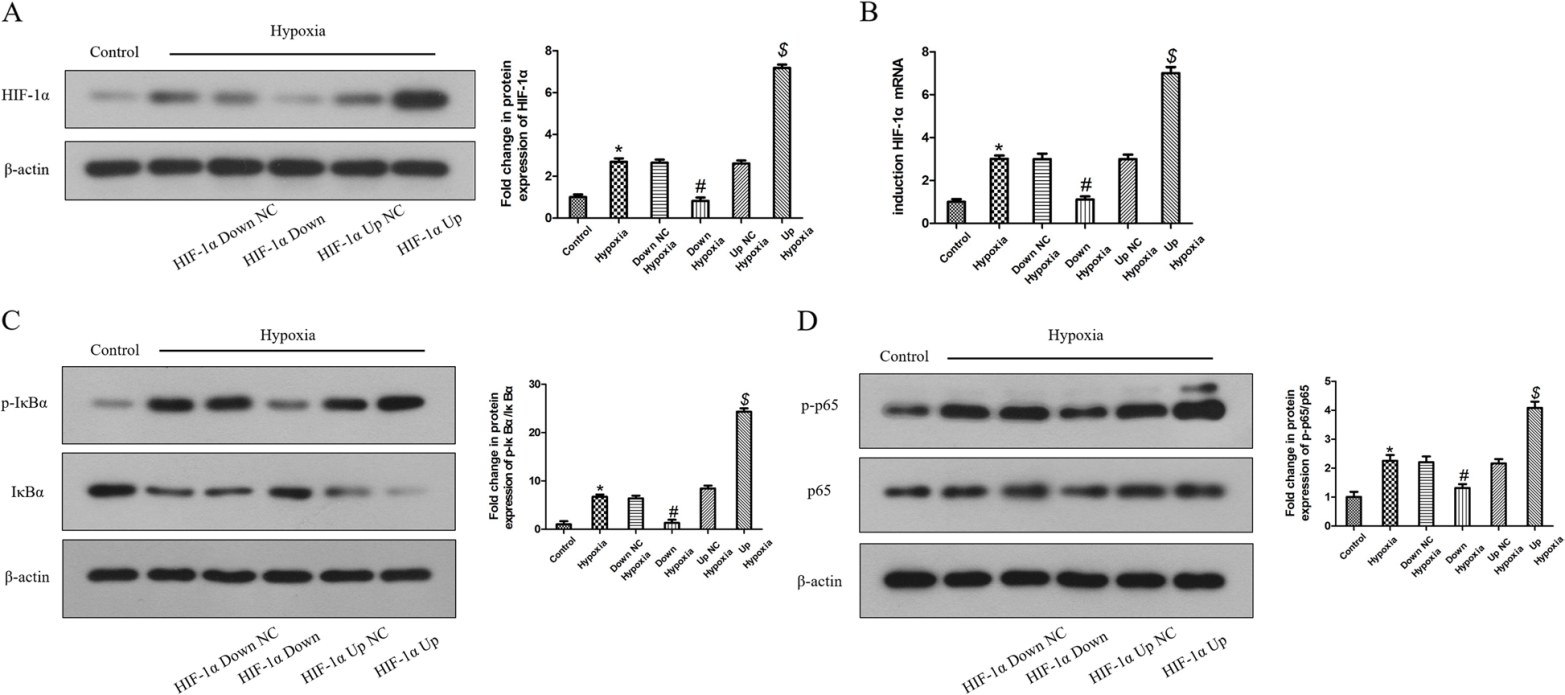
Effects of HIF-1α on hypoxia-induced NF-κB signaling pathway in HDPFs. HDPFs induced by hypoxia for the indicated time with or without HIF-1α downregulation or upregulation. The protein levels of HIF-1α, IκBα, p-IκBα, p65, p-p65 and β-actin was analyzed by western blotting **A**, **C**, **D**. The mRNA expression of HIF-1α was analyzed by qRT-PCR **B**. Statistical analysis was performed using one-way ANOVA with Tukey's Multiple Comparison Test. *P < 0.05 compared with the control group. #*P* < 0.05 compared with the hypoxia-induced group and the Down NC hypoxia-induced group. $*P* < 0.05 compared with the hypoxia-induced group and Up NC hypoxia-induced group. Full-length blots/gels are presented in Supplementary Fig. 6–8

**Fig. 5:**
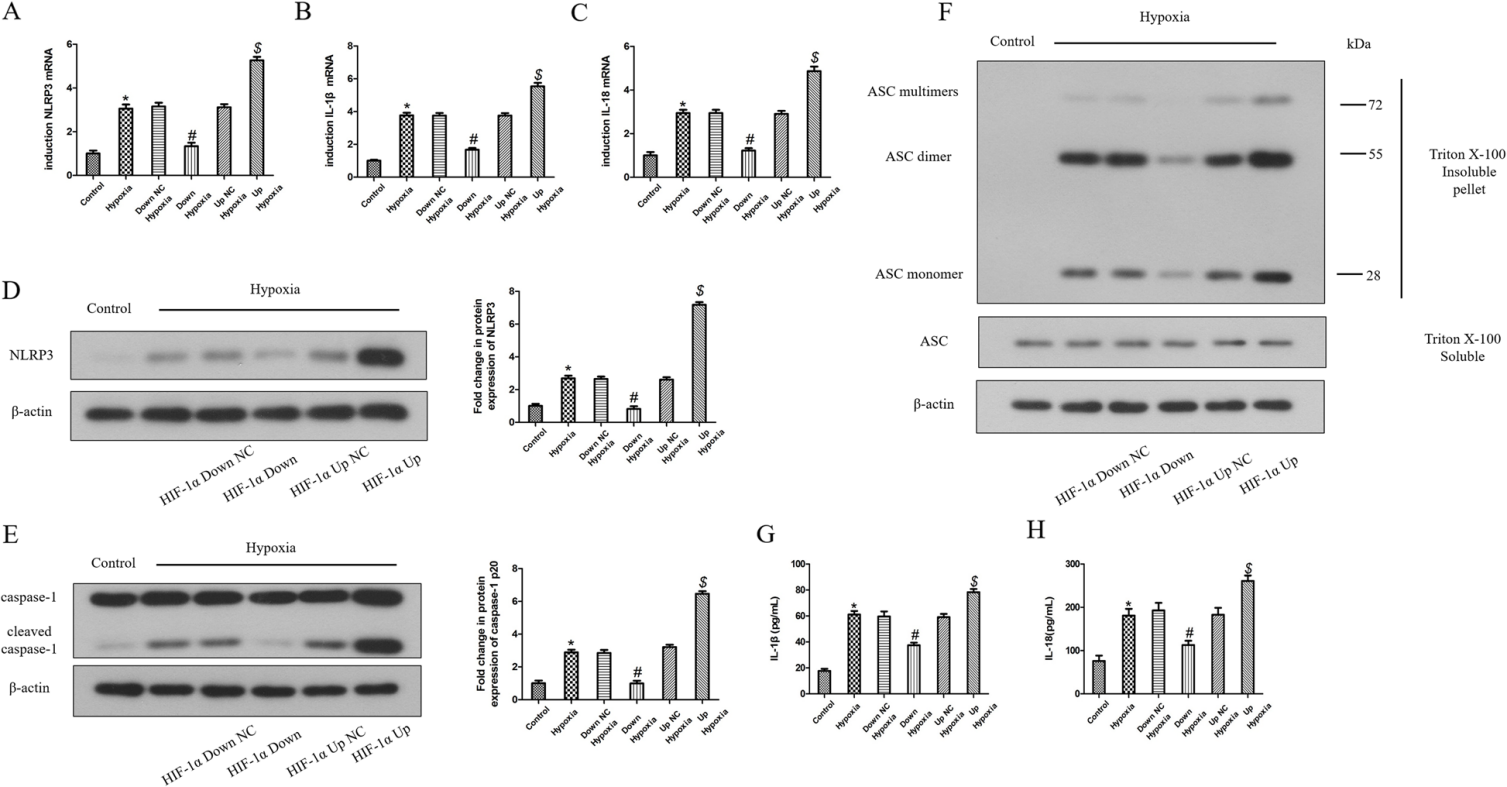
Effects of HIF-1α on hypoxia-induced NLRP3 inflammasome pathway in HDPFs. HDPFs induced by hypoxia for the indicated time with or without HIF-1α downregulation or upregulation. The mRNA and protein expression of NLRP3, caspase-1, ASC, IL-1β and IL-18 were analyzed by qRT-PCR **A***-***C**, western blotting **D-F** and ELISA **G**, **H**, respectively. Statistical analysis was performed using one-way ANOVA with Tukey's Multiple Comparison Test. *P < 0.05 compared with the control group. #*P* < 0.05 compared with the hypoxia-induced group and the Down NC hypoxia-induced group. $*P* < 0.05 compared with the hypoxia-induced group and Up NC hypoxia-induced group. Full-length blots/gels are presented in Supplementary Fig. 9–11

Lentiviral transfection was used to overexpress or knockdown HIF-1α in HDPFs. The mRNA and protein level of HIF-1α in the overexpression group or knockdown group were significantly upregulated or downregulated compared to that of their corresponding NC vector at 12 h of hypoxia, respectively (Fig. 4A, B). Twelve hours of hypoxia upregulated the p-IκBα and p-p65 protein levels (Fig. 4C, D), the ASC oligomerization (Fig. 5F), NLRP3, IL-1β and IL-18 mRNA expression (Fig. 5A-C), NLRP3 and caspase-1 p20 protein expression (Fig. 5D, E), as well as secretion of IL-1β and IL-18 (Fig. 5G, H). Overexpression of HIF-1α further increased the protein levels of p-IκBα and p-p65 (Fig. 4C, D), NLRP3, IL-1β and IL-18 mRNA expression (Fig. 5A-C), NLRP3 and caspase-1 p20 protein expression (Fig. 5D, E), secretion of IL-1β and IL-18 (Fig. 5G, H), and promoted ASC oligomerization (Fig. 5F). In comparison, knockdown of HIF-1α decreased the p-IκBα and p-p65 protein levels (Fig. 4C, D) and correspondingly inhibited ASC oligomerization and downregulated the NLRP3, IL-1β and IL-18 mRNA expression (Fig. 5A-C), NLRP3 and caspase-1 p20 protein expression (Fig. 5D, E), and secretion of IL-1β and IL-18 (Fig. 5G, H).

### The regulatory effect of HIF-1α on ASC oligomerization and subsequent NLRP3/CASP1 inflammasome pathway activation via the NF-κB signaling pathway in HDPFs induced by LPS and hypoxia

**Fig. 6:**
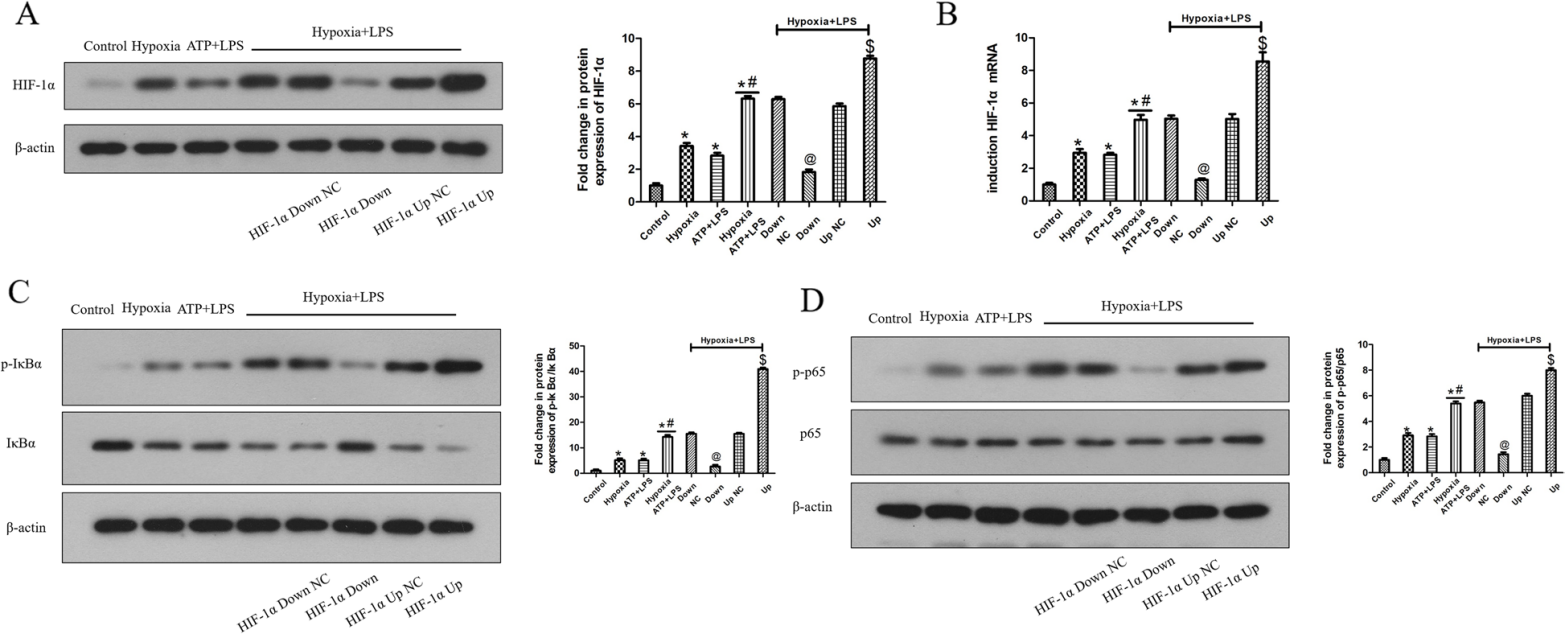
The regulation of HIF-1α on the NF-κB signaling in HDPFs induced by LPS and hypoxia. HDPFs were induced by ATP plus LPS or LPS plus hypoxia for the indicated time with or without HIF-1α downregulation or upregulation. The protein levels of HIF-1α, IκBα, p-IκBα, p65, p-p65 and β-actin was analyzed by western blotting **A**, **C**, **D**. The mRNA expression of HIF-1α was analyzed by qRT-PCR **B**. Statistical analysis was performed using one-way ANOVA with Tukey's Multiple Comparison Test. *P < 0.05 compared with the control group. #*P* < 0.05 compared with the hypoxia-induced group and the Down NC hypoxia-induced group. $*P* < 0.05 compared with the hypoxia-induced group and Up NC hypoxia-induced group. Full-length blots/gels are presented in Supplementary Fig. 12–14

**Fig. 7:**
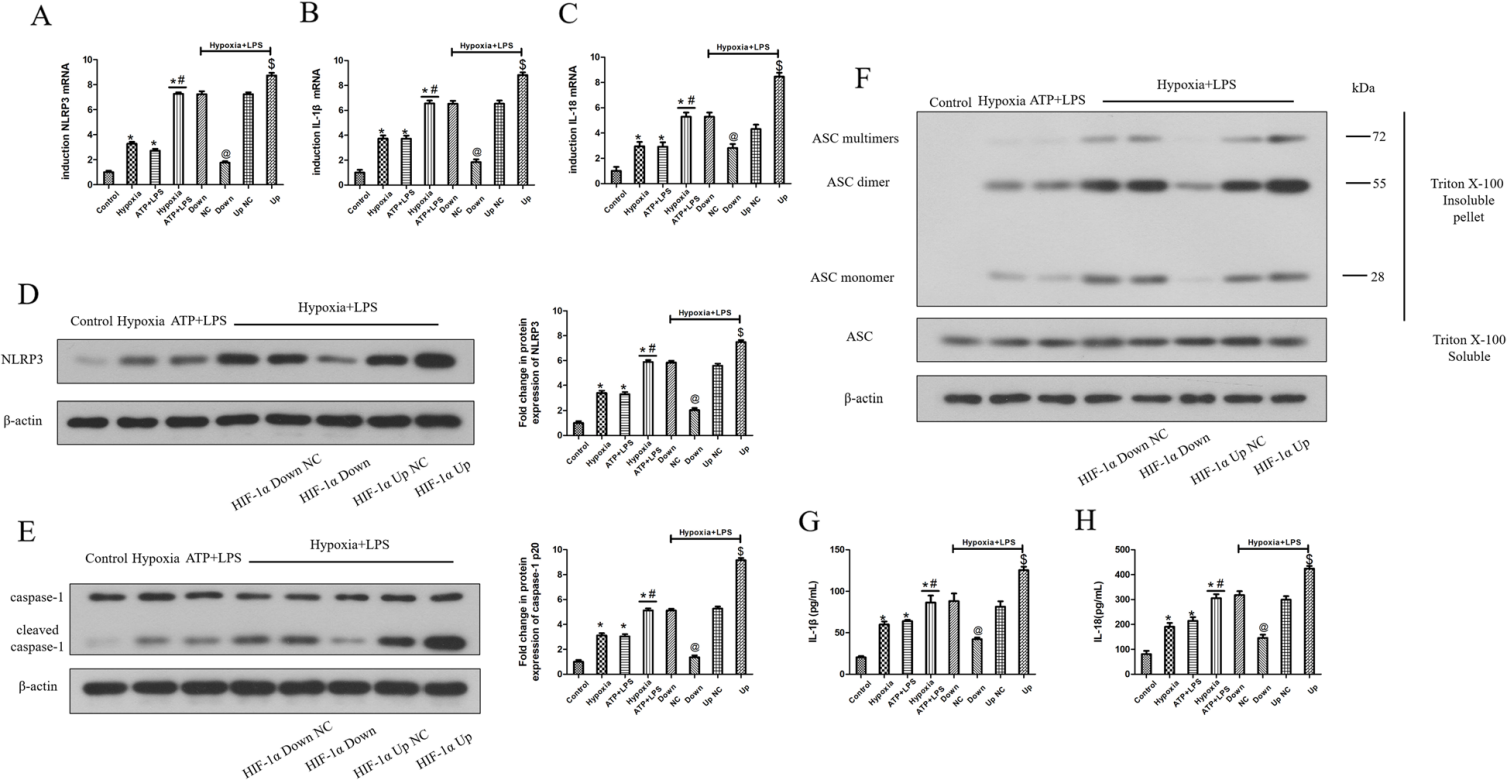
The regulation of HIF-1α on the ASC oligomerization and subsequent NLRP3 inflammasome pathway in HDPFs induced by LPS and hypoxia. HDPFs were induced by ATP plus LPS or LPS plus hypoxia for the indicated time with or without HIF-1α downregulation or upregulation. The mRNA and protein expression of NLRP3, caspase-1, ASC, IL-1β and IL-18 were analyzed by qRT-PCR **A-C**, western blotting **D-F** and ELISA **G**, **H**, respectively. Statistical analysis was performed using one-way ANOVA with Tukey's Multiple Comparison Test. *P < 0.05 compared with the control group. #*P* < 0.05 compared with the hypoxia-induced group and the Down NC hypoxia-induced group. $*P* < 0.05 compared with the hypoxia-induced group and Up NC hypoxia-induced group. Full-length blots/gels are presented in Supplementary Fig. 15–17

Compared to the ATP plus LPS-induced group and hypoxia-induced group, LPS and hypoxia exerted synergetic effects on the upregulation of the expression of HIF-1α (Fig. 6A, B), p-IκBα and p-p65 (Fig. 6C, D), the promotion of ASC oligomerization (Fig. 7F) and upregulation of NLRP3, IL-1β and IL-18 mRNA expression (Fig. 7A-C), NLRP3 and caspase-1 p20 protein expression (Fig. 7D, E), and secretion of IL-1β and IL-18 (Fig. 7G, H). Additionally, overexpression of HIF-1α further significantly increased LPS- and hypoxia-induced p-IκBα and p-p65 levels (Fig. 6C, D) and promoted ASC oligomerization (Fig. 7F), and upregulated NLRP3, IL-1β and IL-18 mRNA expression (Fig. 7A-C), NLRP3 and caspase-1 p20 protein expression (Fig. 7D, E), and secretion of IL-1β and IL-18 (Fig. 7G, H). In contrast, knockdown of HIF-1α decreased LPS- and hypoxia-induced p-IκBα and p-p65 (Fig. 6C, D) and inhibited ASC oligomerization (Fig. 7F), and downregulated NLRP3, IL-1β and IL-18 mRNA expression (Fig. 7A-C), NLRP3 and caspase-1 p20 protein expression (Fig. 7D, E), and secretion of IL-1β and IL-18 (Fig. 7G, H).

## Discussion

Dental pulp contains heterogeneous cell populations, including odontoblasts, fibroblasts, inflammatory and immune cells and stem cells, which are responsible for its maintenance, defense and repair [26]. Odontoblasts are the first cells to encounter bacterial infection or their toxins because of their location at the periphery of the pulp [26]. As the infection advances, cells deeper in the pulp core, including fibroblasts, endothelial cells, immune cells and stem cells, are also involved in defense responses [27]. HDPFs, which are the predominant cells within dental pulp, play a central role in pulpal inflammation by secreting proinflammatory cytokines in response to injuries or caries-related microorganisms [28]. It has been demonstrated that HDPFs recognize pathogen-associated molecular patterns (PAMPs) produced by invading bacteria or damage-associated molecular patterns (DAMPs) produced by the host by expressing a range of pattern recognition receptors (PRRs), thus initiating inflammatory/immune responses in pulpal tissue [18, 19, 29]. A previous study by our lab showed that as dental caries advanced, the odontoblast layers were disrupted, dental pulp cells displayed extensive staining for HIF-1α and NLRP3, and the upregulation of HIF-1α was associated with the activation of the NLRP3/CASP1 inflammasome pathway in irreversible pulpitis in vivo [21]. These findings provide evidence for the key role of the NLRP3 inflammasome in the link between hypoxia and pulpal inflammation. However, whether hypoxia alone can directly activate the NLRP3 inflammasome and its underlying molecular mechanisms remain to be explored.

In the absence of any microorganism infection, inflammatory responses occur as a result of trauma, ischemia‒reperfusion injury or chemically induced injury [30]. This type of inflammation is referred to as sterile inflammation [30]. Similar to infection-induced inflammation, sterile inflammation is characterized by the recruitment of immune cells and the secretion of proinflammatory cytokines and chemokines, especially tumor necrosis factor (TNF-α) and IL­1β [30]. It has been reported that sterile inflammation can occur in pulpal tissue and periodontal tissue caused by dental trauma or orthodontic force application [31]. The effect of orthodontic force induces circulatory disturbances in the periodontal tissue and in the pulpal tissue, leading to the upregulation of proinflammatory and angiogenic genes that are associated with the hypoxic microenvironment in dental pulp [31, 32]. In the present study, the effect of hypoxia alone on HDPFs was evaluated to simulate sterile pulpal inflammation. We found that hypoxia activated NF-κB signaling in HDPFs, as evidenced by the downregulation of IκBα, upregulation of p-IκBα and subsequent upregulation of NF-κB p-p65.

ASC oligomerization is a key event in the activation of the NLRP3 inflammasome [17]. Numerous ASC events related to NLRP3 inflammasome activation have been reported [25, 33, 34]. Therefore, ASC oligomerization is a hallmark of NLRP3 inflammasome formation. In this study, we further showed that hypoxia promoted the formation of a dimer or oligomer of ASC and subsequent activation of the NLRP3 inflammasome. In a previous study, we demonstrated that NF-κB activation was involved in the LPS-induced upregulation of NLRP3 and IL-1β in HDPFs [20]. Thus, we hypothesize that hypoxia induces ASC oligomerization and subsequent activation of the NLRP3/CASP1 inflammasome pathway via NF-κB activation in HDPFs. Previous studies have suggested that HIF-1α and NF‐κB signaling play pivotal roles in the molecular link between hypoxia and inflammation [23]. Jordi et al. reported that chromatin immunoprecipitation (ChIP) in LPS-stimulated macrophages revealed that NF-κB p65 (RelA) is recruited to the HIF-1α promoter, which contains a classical kB site at 2197/2188 base pairs, conserved between mice and humans [35]. In this study, we found that HIF-1α was faintly observed in HDPFs that were cultured in the normoxic conditions, but HIF-1α protein expression was strongly increased, and it translocated to the nucleus and was expressed in both the cytoplasm and nucleus in HDPFs that were cultured under hypoxic conditions. Experiments with up- and downregulated HIF-1α expression revealed that HIF-1α acted as a positive regulator of ASC oligomerization and subsequent NLRP3/CASP1 inflammasome pathway activation via NF-κB signaling in HDPFs cultured under hypoxic conditions.

During the progression of caries-related pulpitis, bacterial infection is naturally accompanied by a hypoxic microenvironment. Both factors are highly connected and important and shape the pulpitis microenvironment. Gram-negative bacteria play a central role in deep caries and pulpitis. As a major component of the membrane of gram-negative bacteria, LPS has been shown to be responsible for pulpal infection and can induce inflammatory/immune responses in pulp tissue [36]. Additionally, LPS can promote the accumulation of HIF-1α protein in macrophages through transcriptional and translational activation, which is independent of hypoxia-induced HIF-1α protein stabilization [37]. Therefore, the combined effects of a hypoxic microenvironment and LPS on HDPFs were evaluated to simulate the caries-related pulpitis microenvironment. We found that LPS could effectively upregulate the expression of HIF-1α. More importantly, LPS and hypoxic conditions have synergistic effects on the promotion of HIF-1α expression, regulating ASC oligomerization and subsequent NLRP3/CASP1 inflammasome pathway activation via NF-κB signaling. These findings indicated that bacterial irritants and a hypoxic microenvironment might be the key factors that aggravate the progression of caries-related pulpitis. Therefore, effectively eliminating bacterial infections and leaving a certain space in the pulp cavity to alleviate the hypoxic microenvironment may be crucial to controlling the progression of caries-related pulpitis and preserving vital pulp. The limitation of the study is that the hypoxic conditions in vitro cannot fully simulate the hypoxic environment of dental pulp inflammation in vivo. More work is required to investigate the correlation between hypoxic microenvironment and pulpitis in vivo, attempting to control the progression of pulpitis and preserve vital pulp by alleviating the hypoxic microenvironment in the pulp cavity or pharmacological interventions targeting HIF-1α.

## Conclusions

In this study, we simulated sterile pulpal inflammation and demonstrated that hypoxic conditions alone could activate the NLRP3/ASC/CASP1 inflammasome pathway via NF-κB signaling in HDPFs. Furthermore, we simulated the caries-related pulpitis microenvironment and revealed that LPS and hypoxic conditions had synergistic effects in promoting the NLRP3/ASC/CASP1 inflammasome pathway activation via NF-κB signaling in HDPFs. Hypoxia-induced HIF-1α, which acted as a positive regulator and was involved in the regulation of NLRP3/ASC/CASP1 inflammasome pathway activation via NF-κB signaling in HDPFs. The finding of a novel functional HIF-1α-NF-κB-NLRP3 axis provides insight into the link between the hypoxic microenvironment and pulpal inflammation and supports strategies to regulate this axis that may be beneficial for the control of pulpal inflammation.

## Supplementary Information

Supplementary Material 1.

## Data Availability

The datasets used and/or analysed during the current study are available from the corresponding author on reasonable request.
