# Supplementary material for: Hypoxia-induced NLRP3 inflammasome activation via the HIF-1α/NF-κB signaling pathway in human dental pulp fibroblasts
Source: BMC Oral Health. 2024 Sep 29;24:1156. doi: 10.1186/s12903-024-04936-w (PMC11441079; doi:10.1186/s12903-024-04936-w)

Supplementary Figure 1: Full-length blots/gels of Figure.2 E.

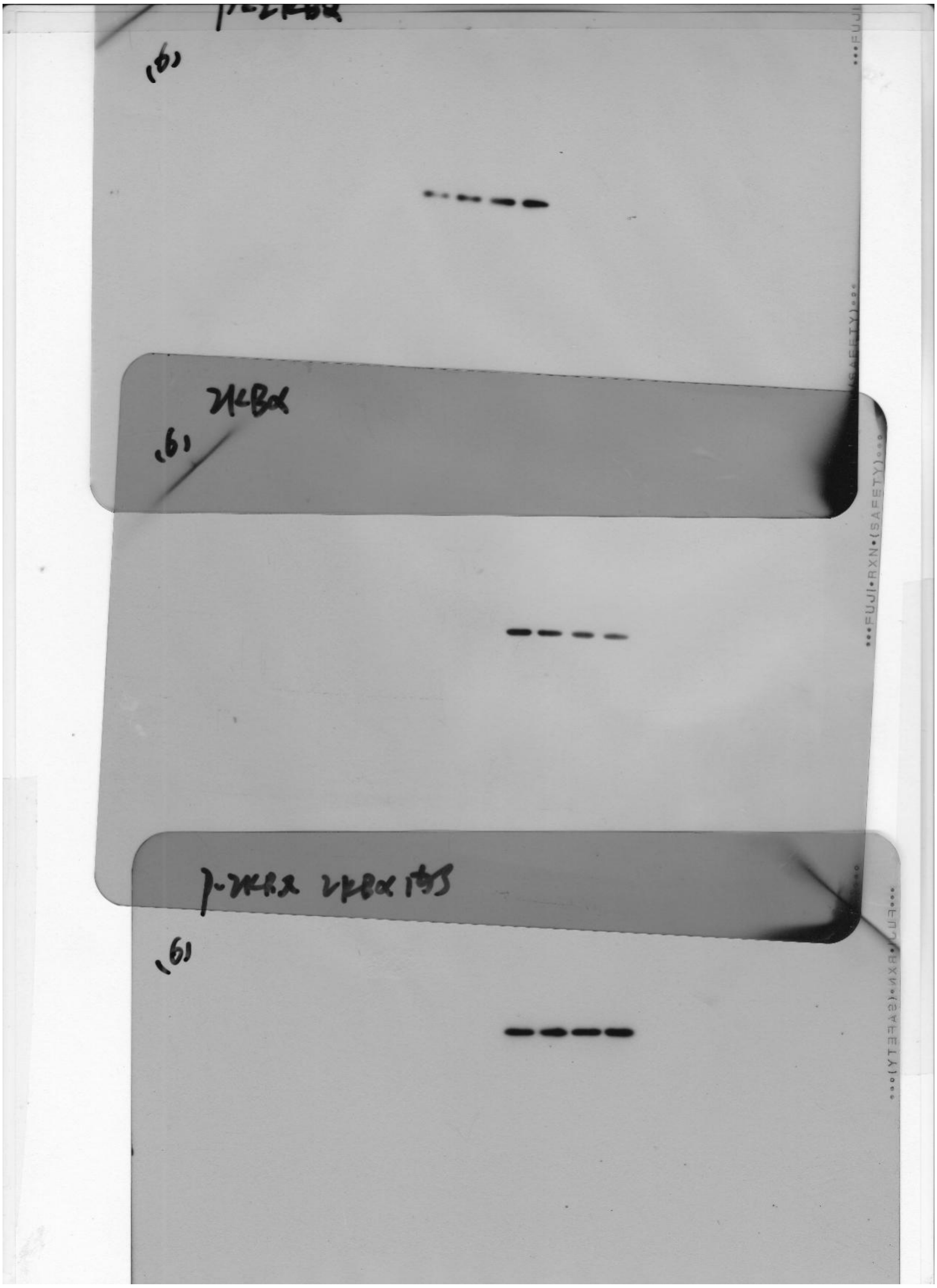

Supplementary Figure 2: Full-length blots/gels of Figure.2 F.

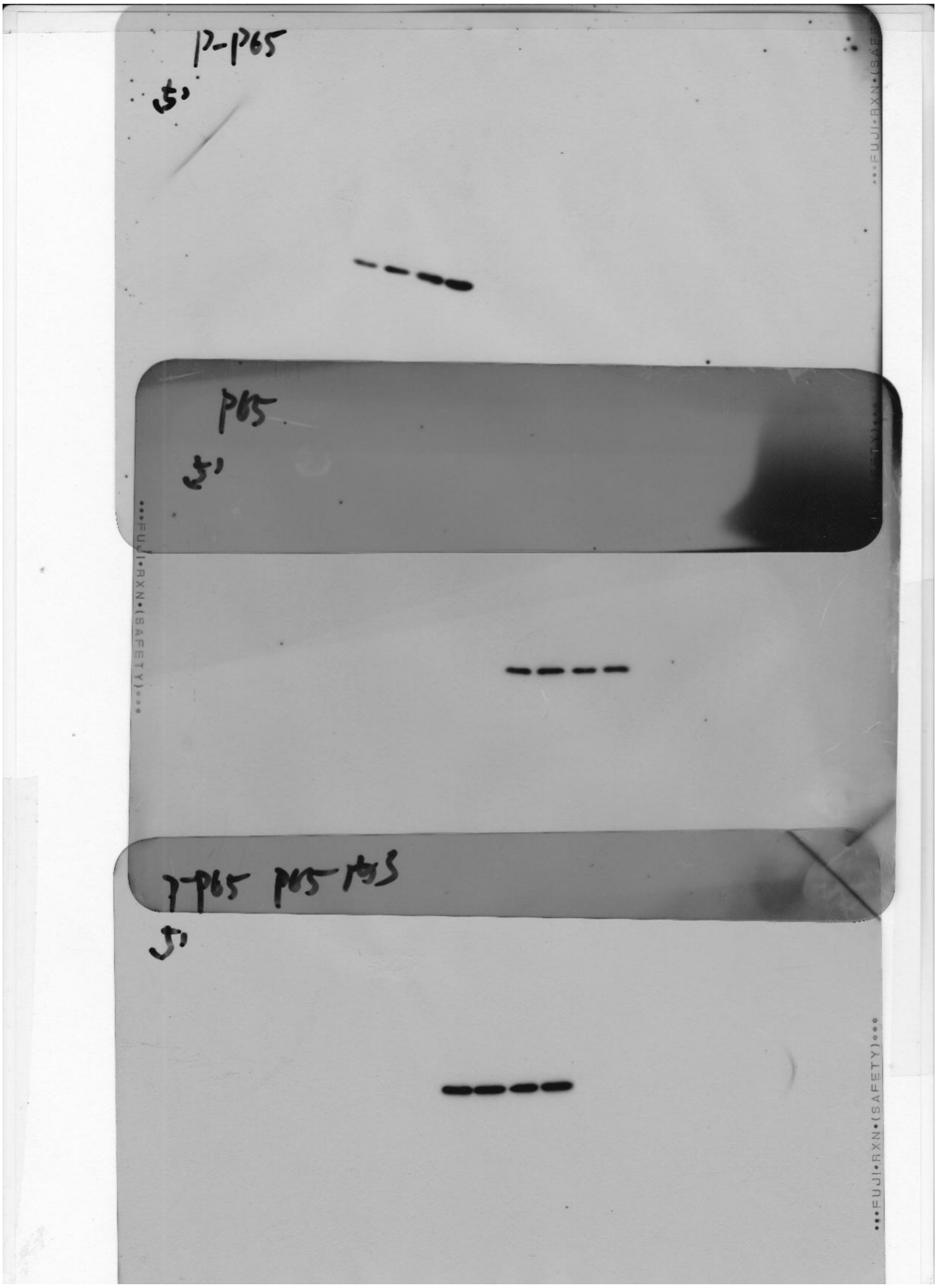

Supplementary Figure 3: Full-length blots/gels of Figure.3 D.

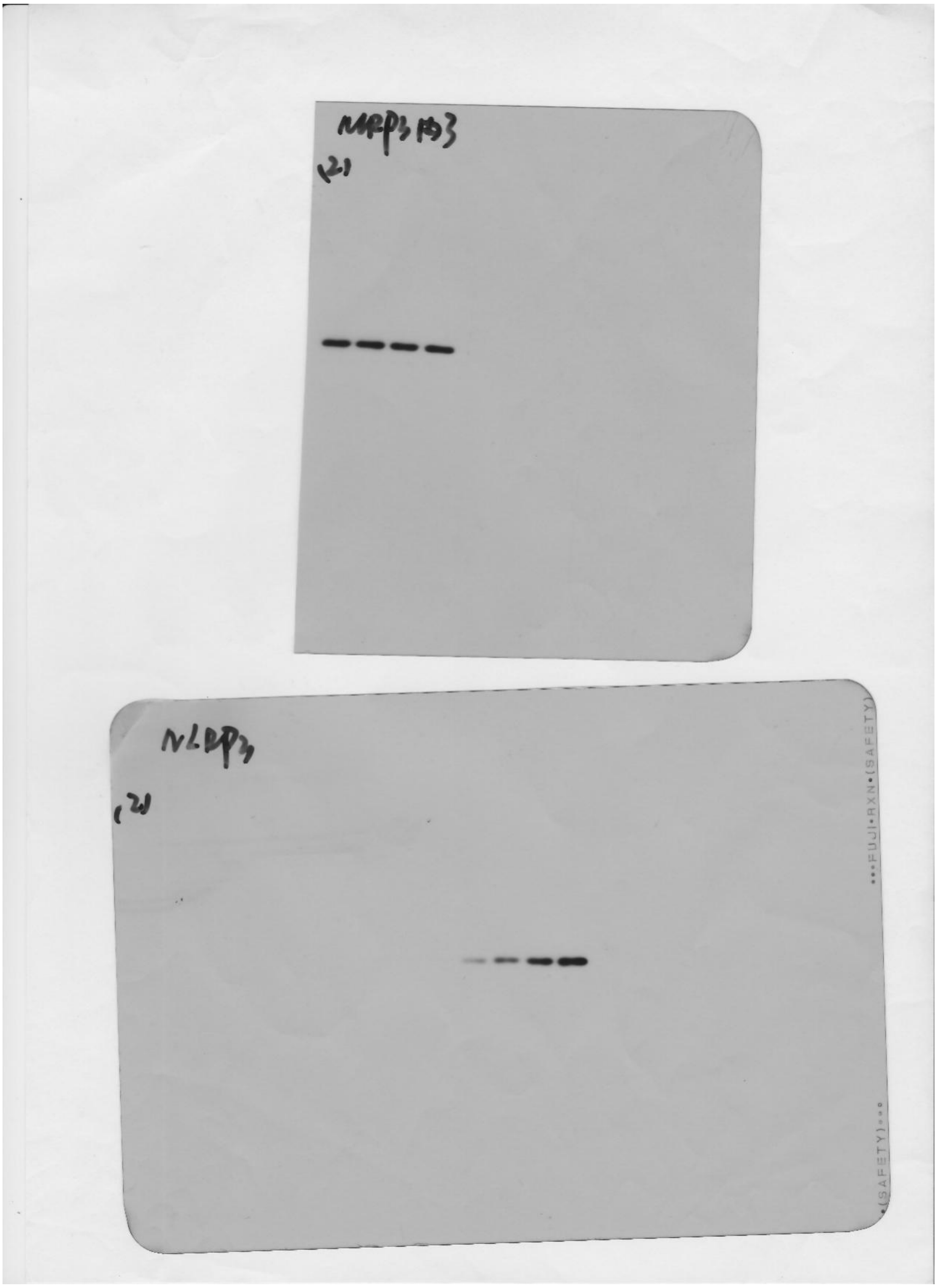

Supplementary Figure 4: Full-length blots/gels of Figure.3 E.

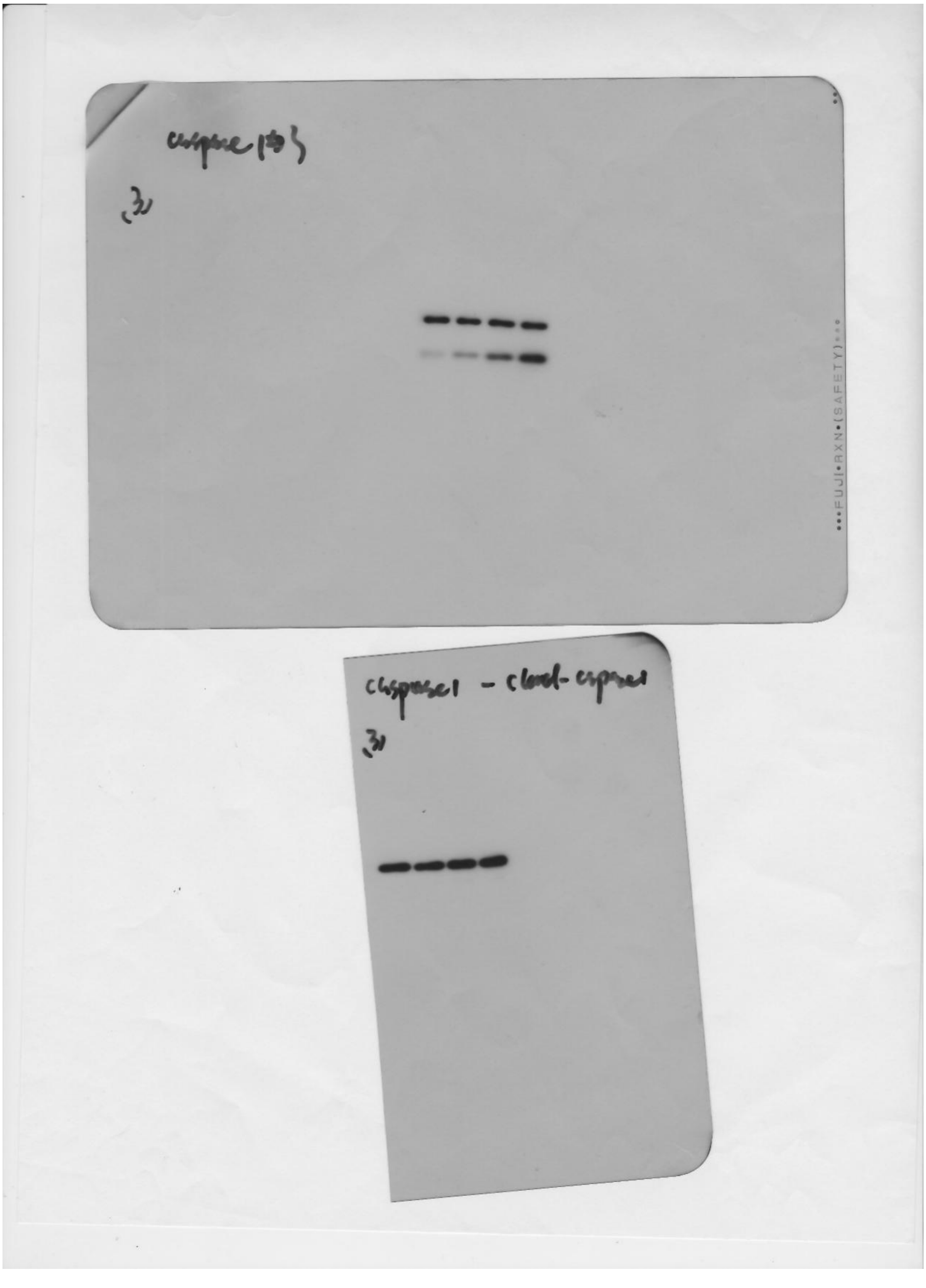

Supplementary Figure 5: Full-length blots/gels of Figure.3 F.

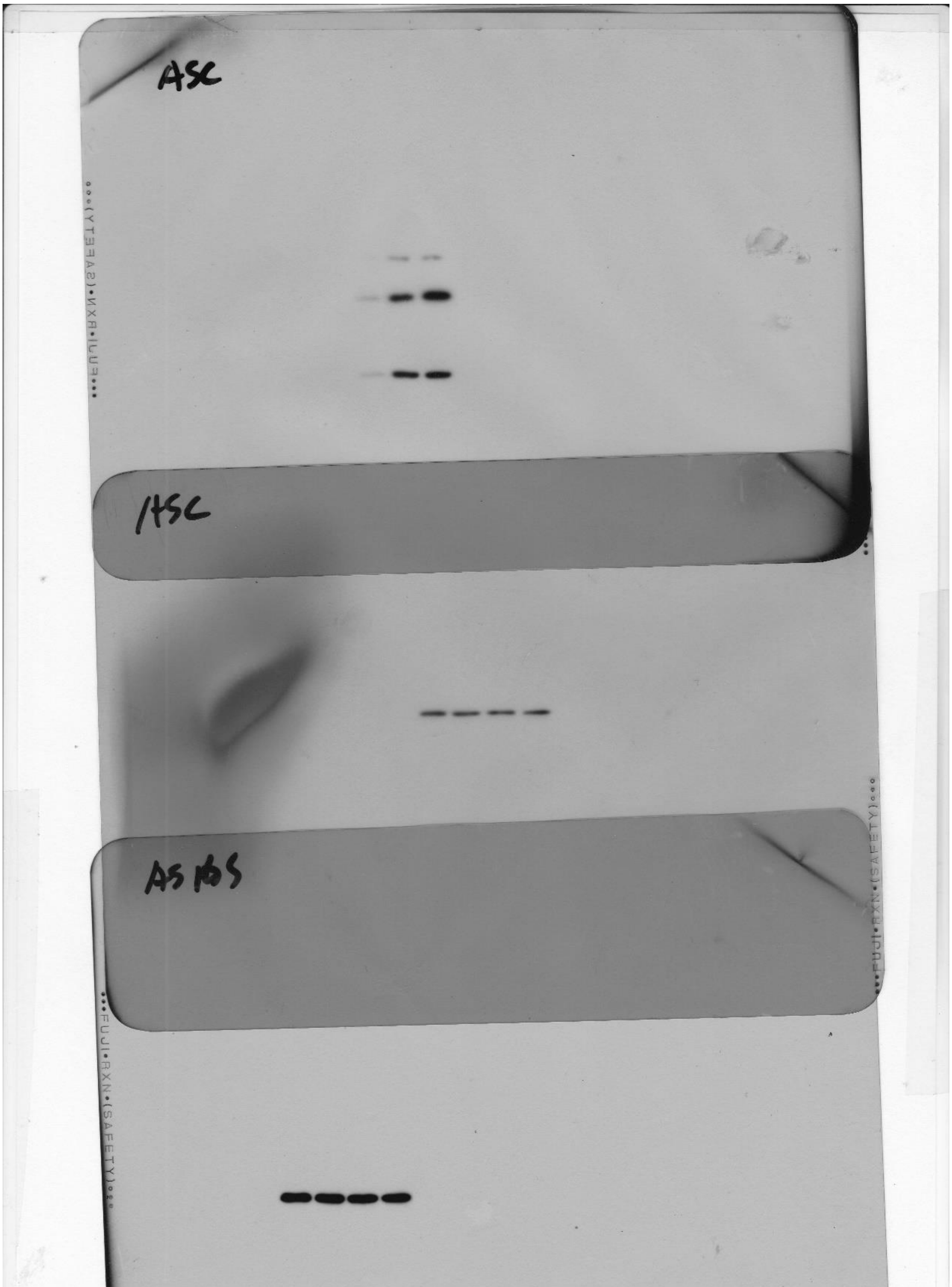

Supplementary Figure 6: Full-length blots/gels of Figure.4 A.

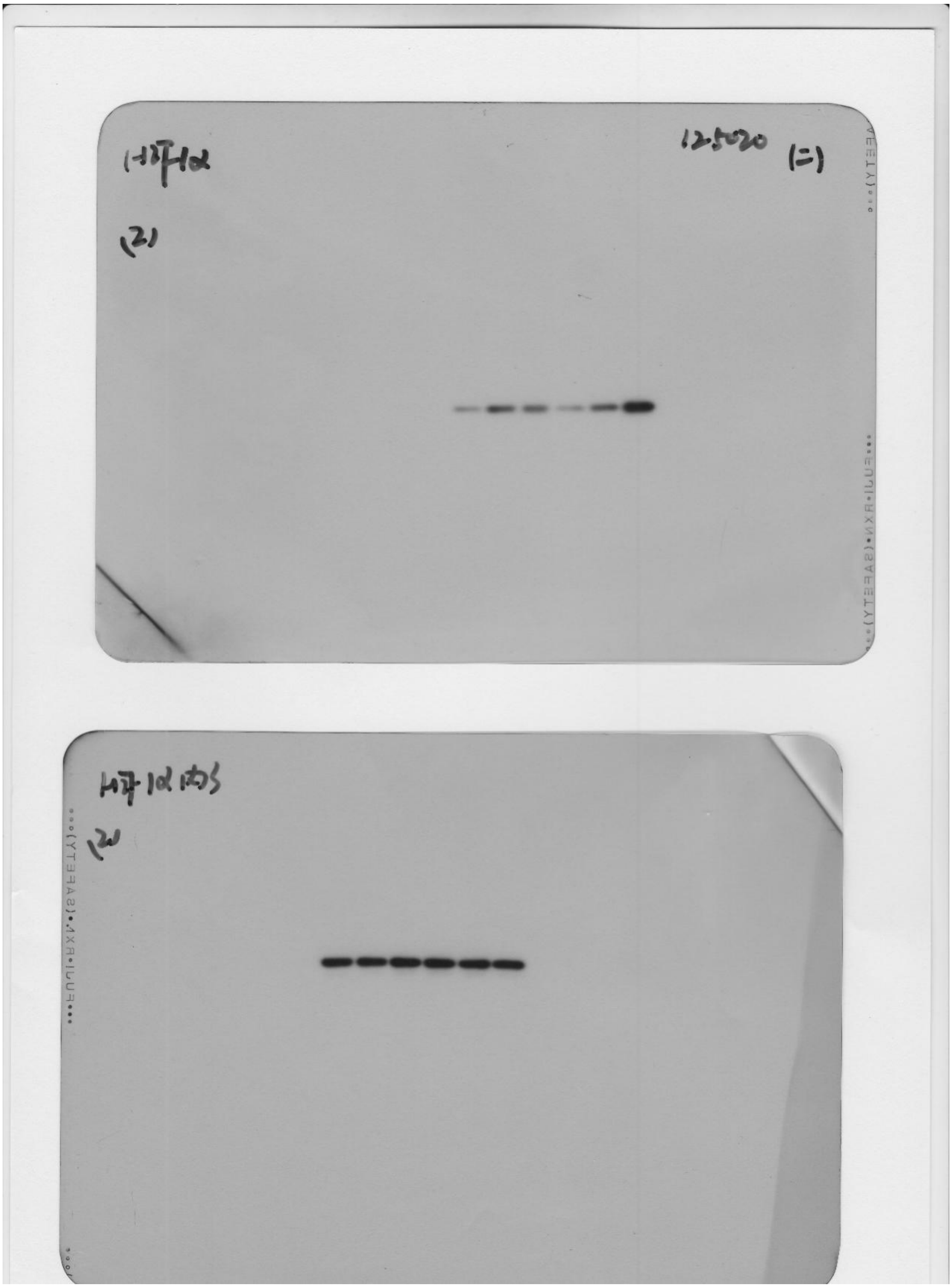

Supplementary Figure 7: Full-length blots/gels of Figure.4 C.

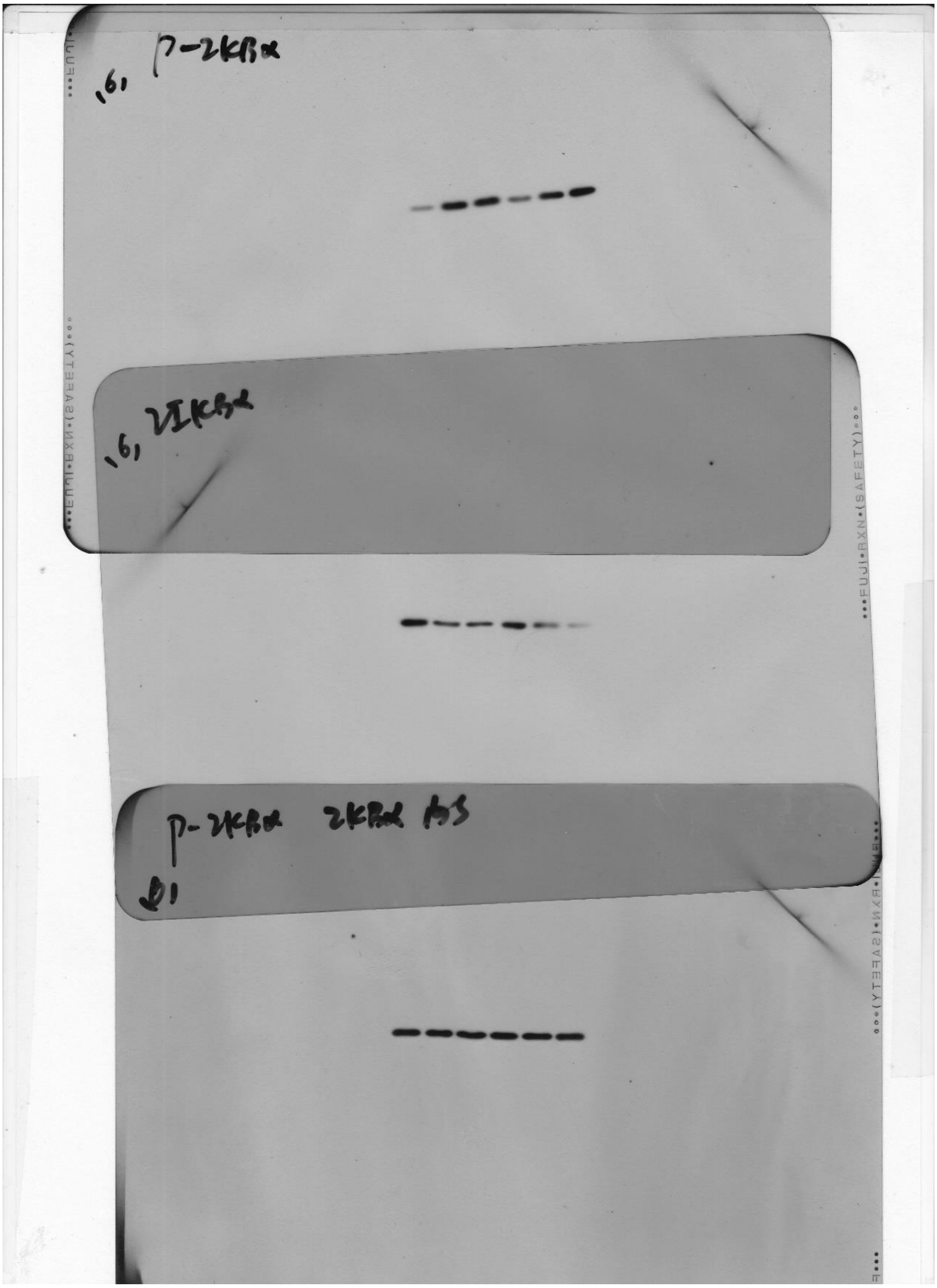

Supplementary Figure 8: Full-length blots/gels of Figure.4 D.

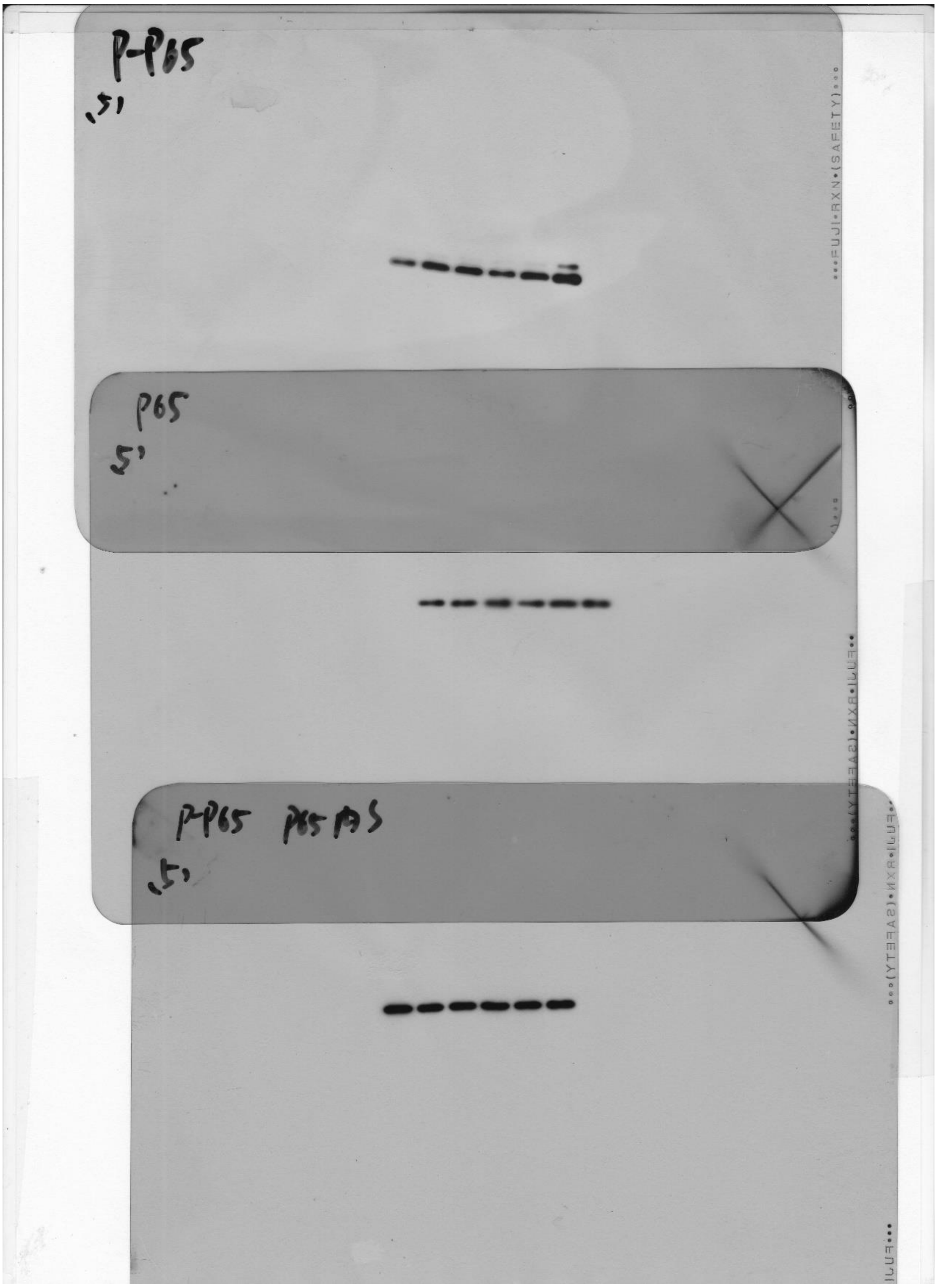

Supplementary Figure 9: Full-length blots/gels of Figure.5 D.

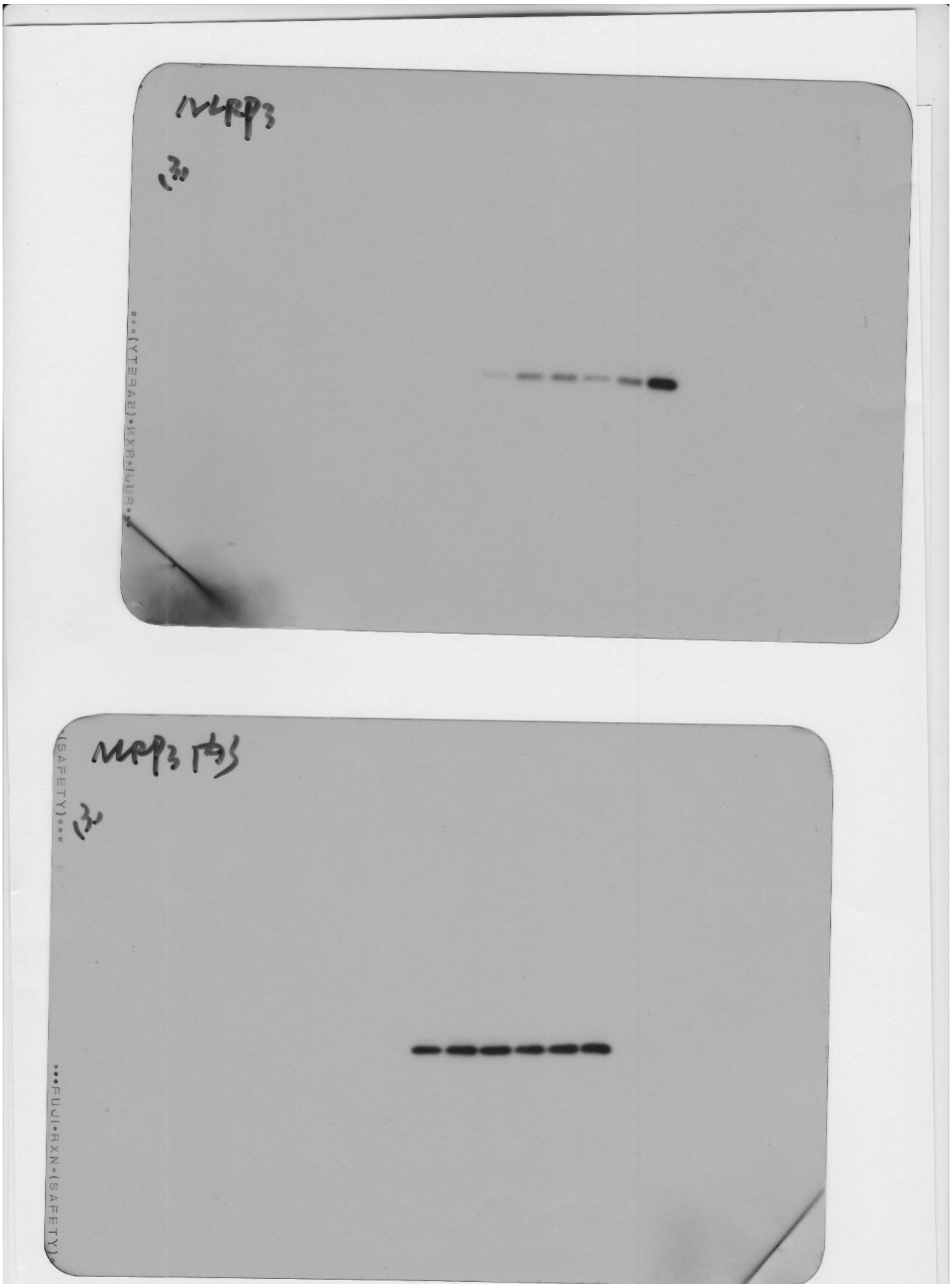

Supplementary Figure 10: Full-length blots/gels of Figure.5 E.

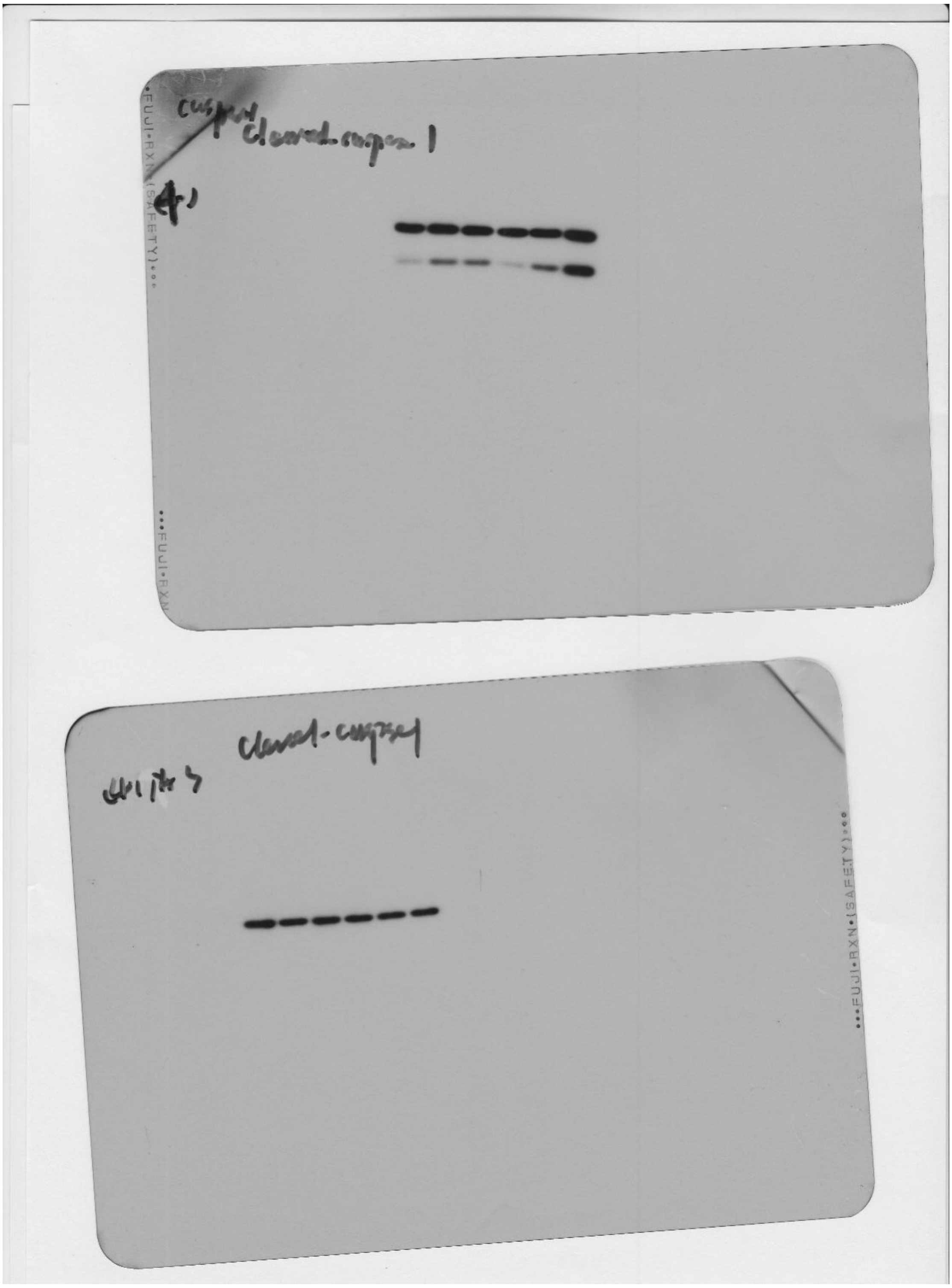

Supplementary Figure 11: Full-length blots/gels of Figure.5 F.

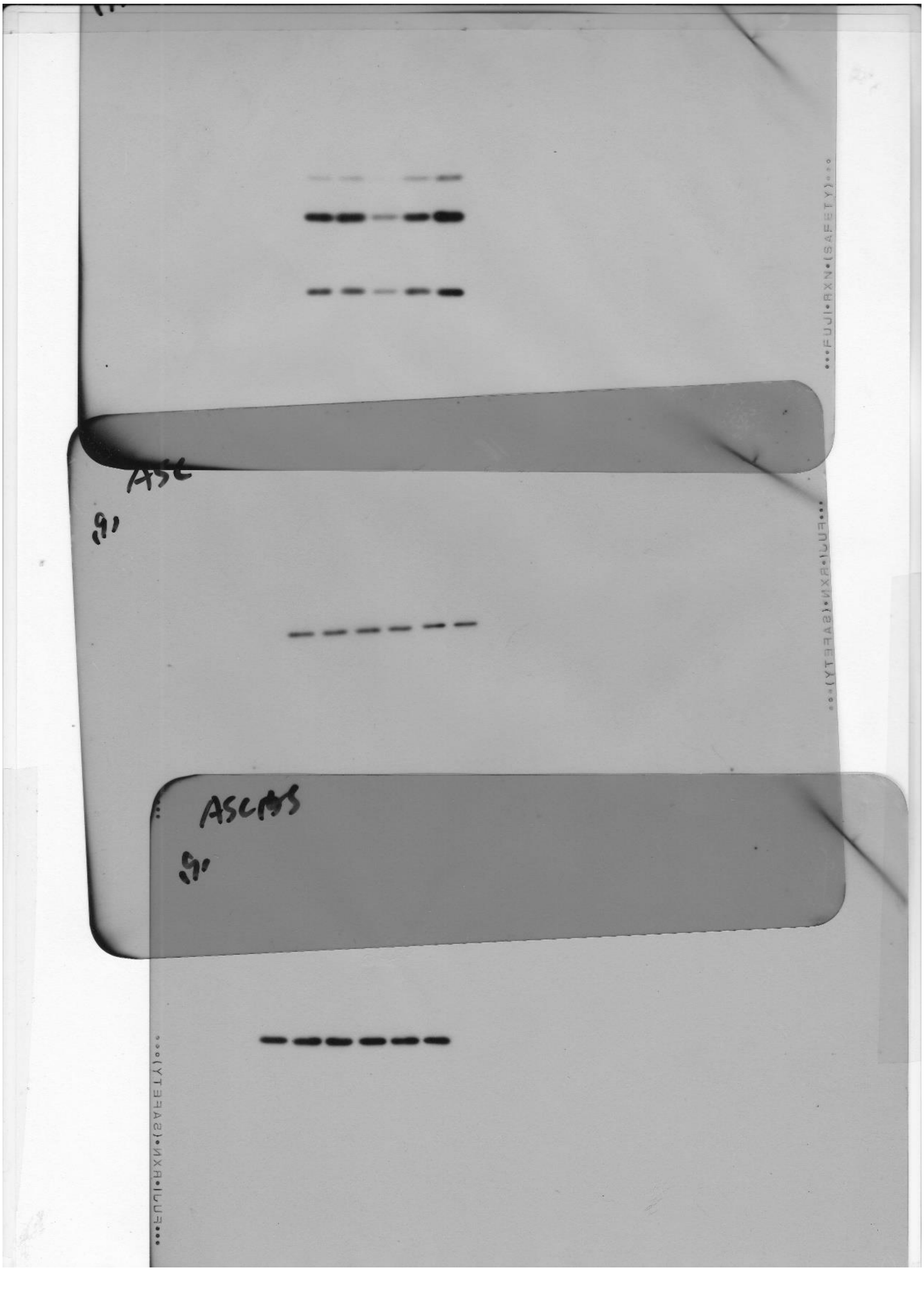

Supplementary Figure 12: Full-length blots/gels of Figure.6 A.

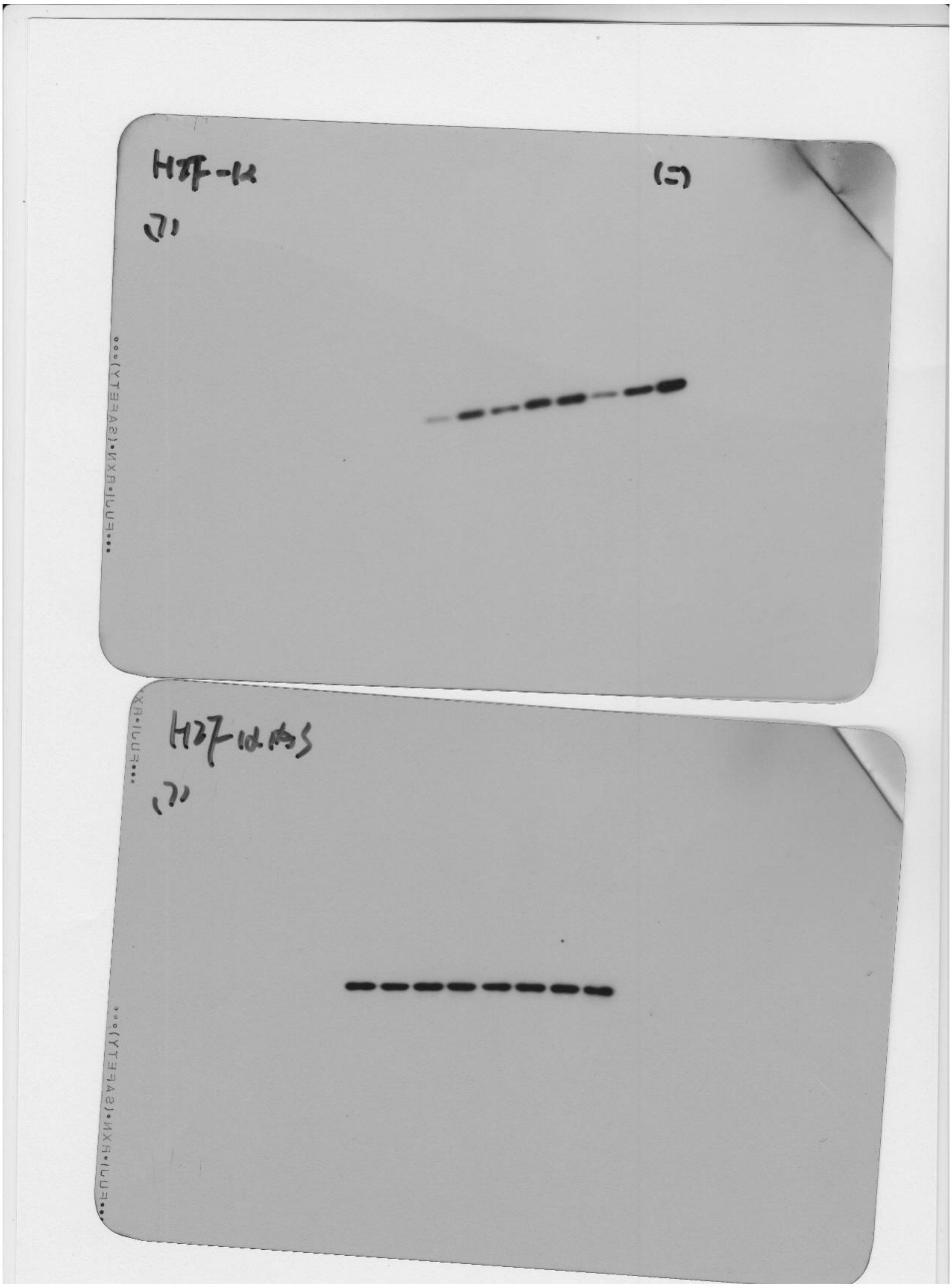

Supplementary Figure 13: Full-length blots/gels of Figure.6 C.

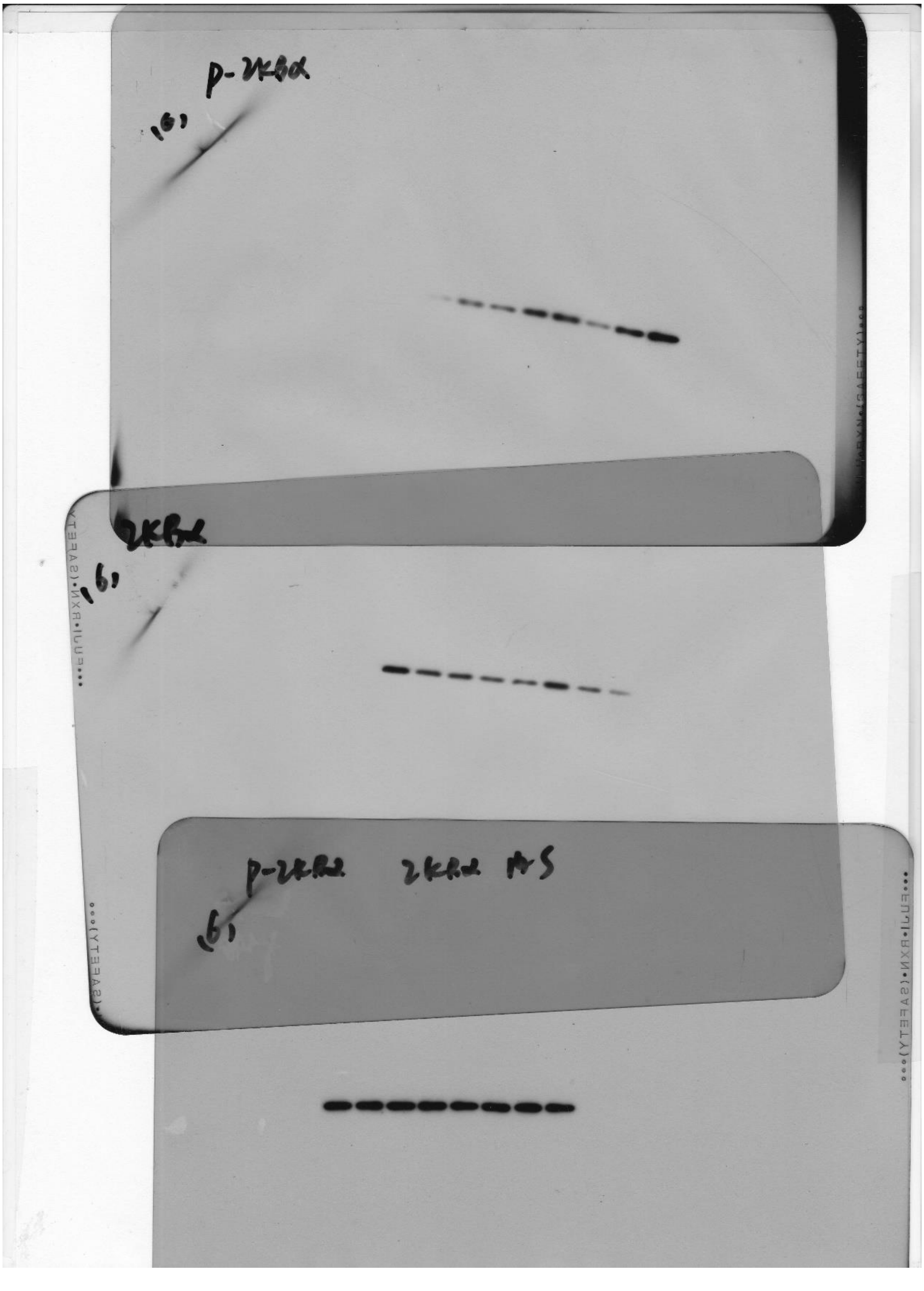

Supplementary Figure 14: Full-length blots/gels of Figure.6 D.

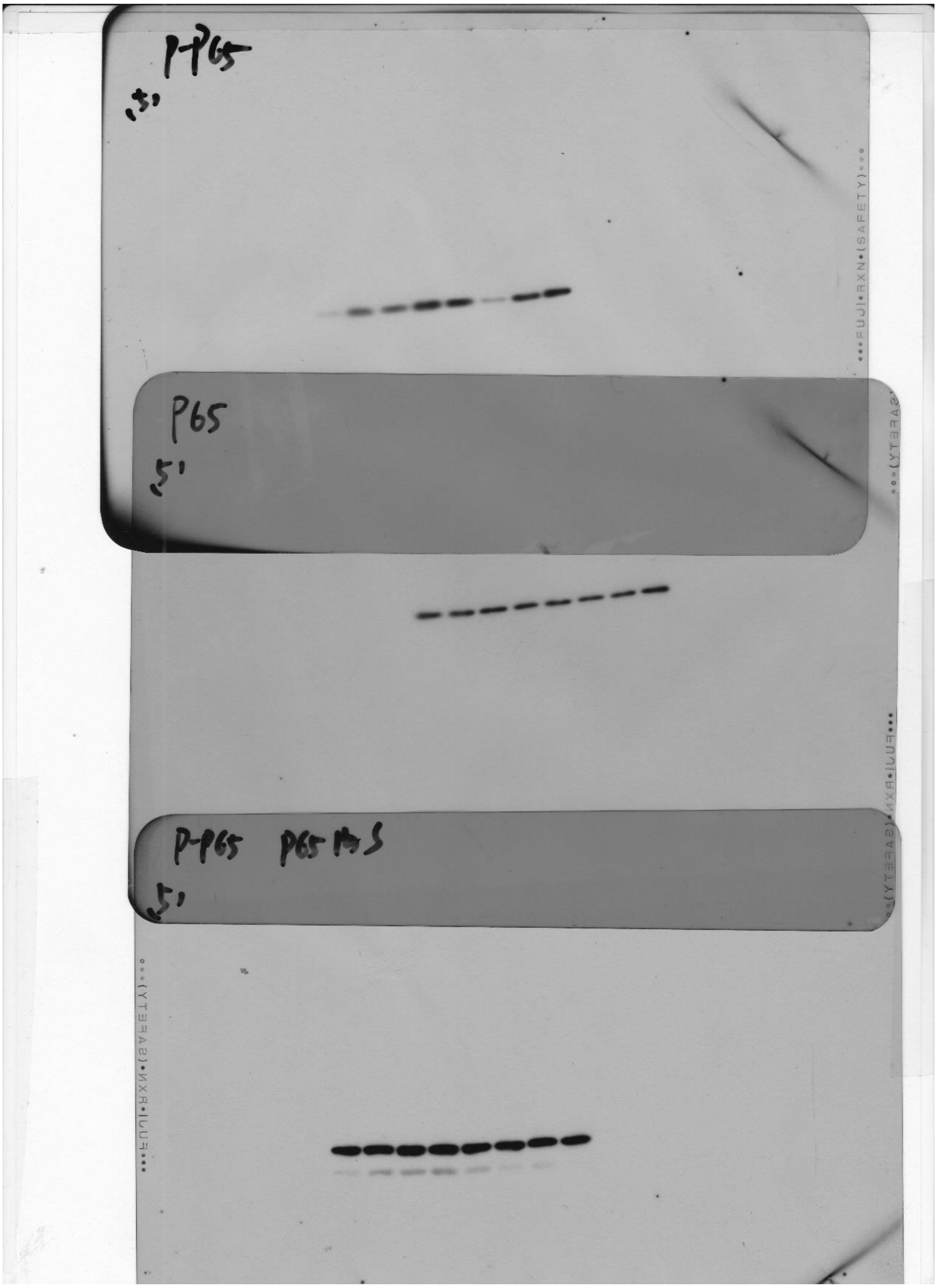

Supplementary Figure 15: Full-length blots/gels of Figure.7 D.

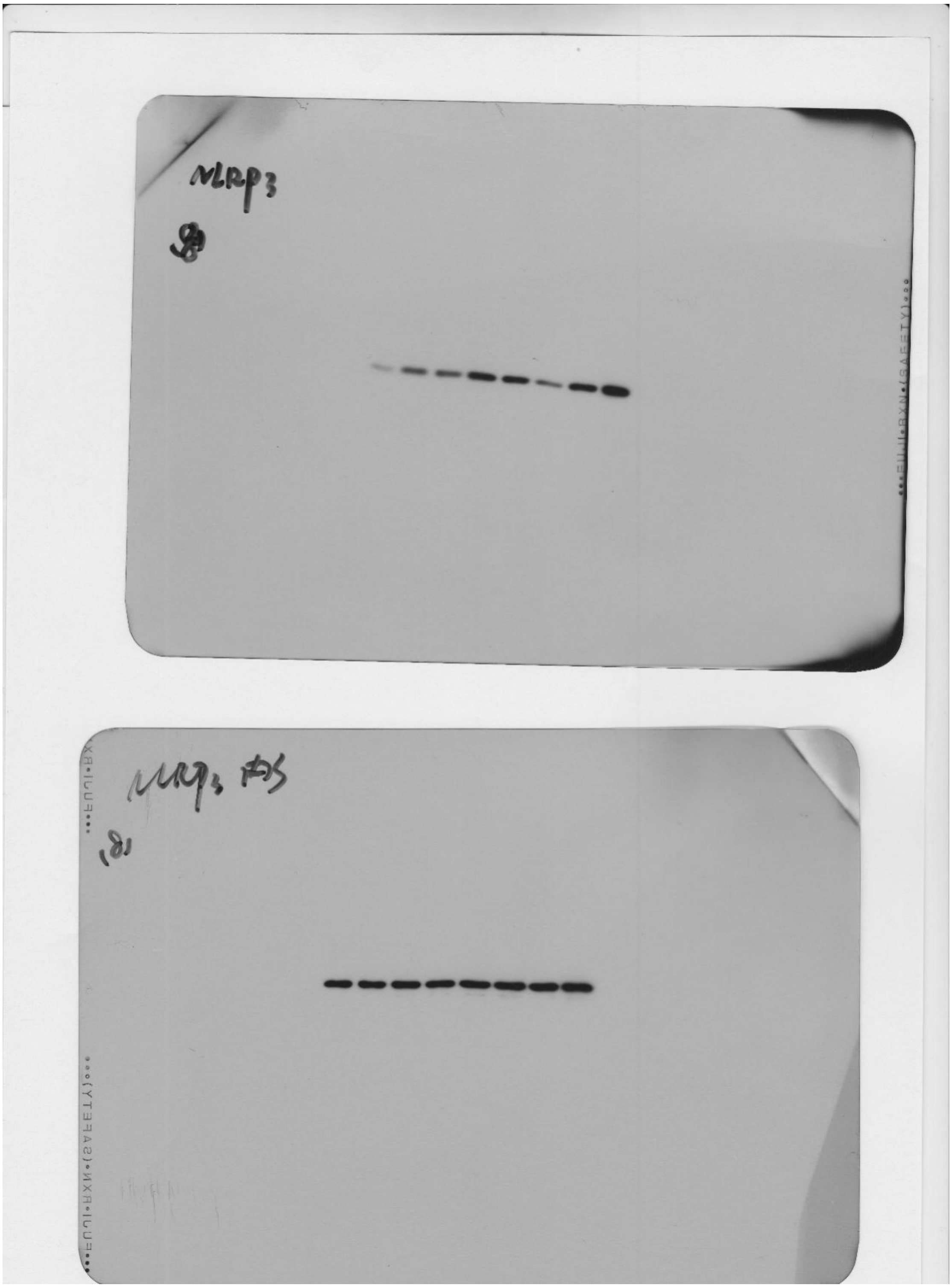

Supplementary Figure 16: Full-length blots/gels of Figure.7 E.

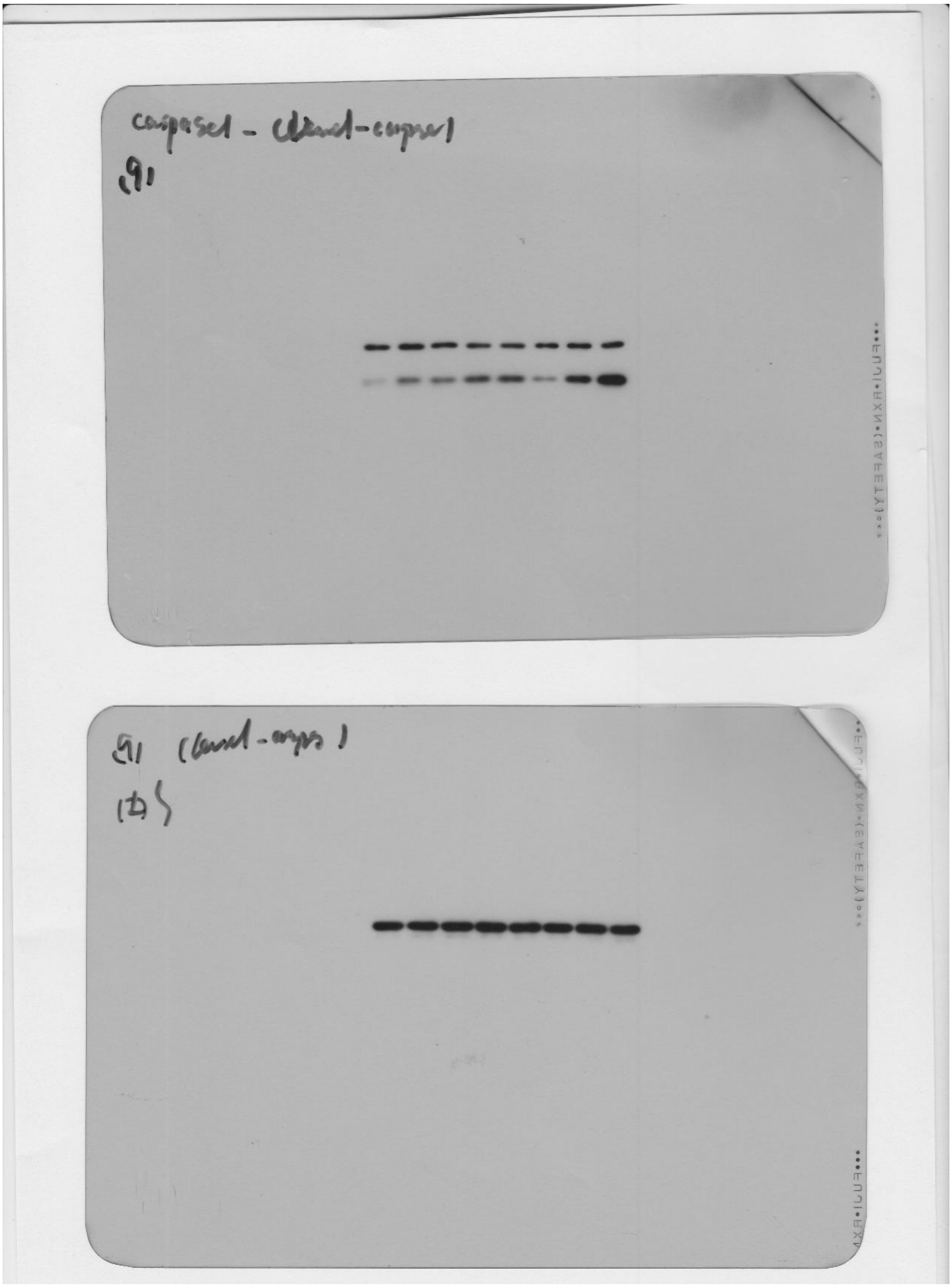

Supplementary Figure 17: Full-length blots/gels of Figure.7 F.

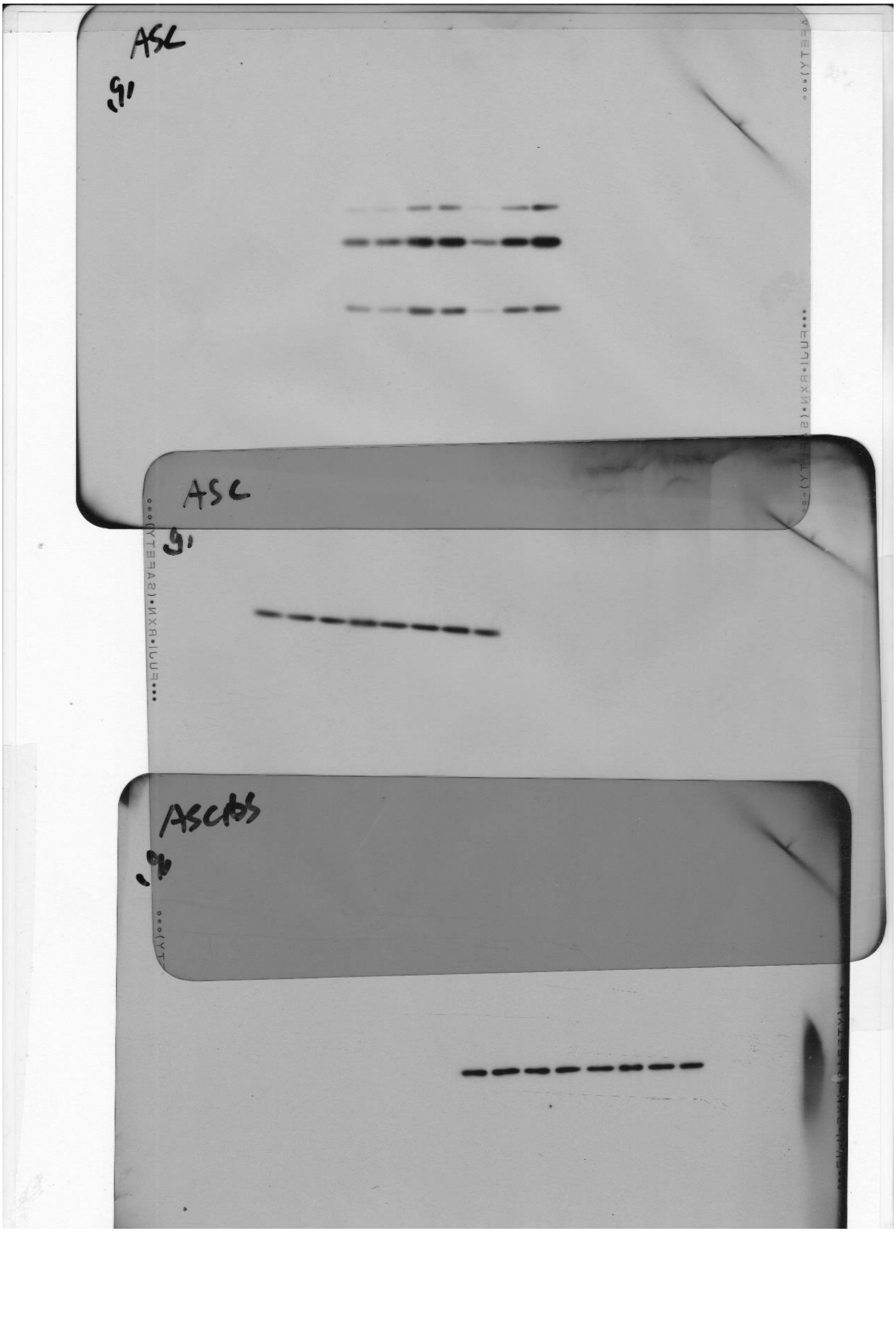

Supplement: Supplementary file 1 — Supplementary Material 1. [file 12903_2024_4936_MOESM1_ESM.pdf]
